# Supplementary figures and images for: Eye movements reflect memory-related theta activity in the human brain
Source: PLoS Biol. 2026 Mar 16;24(3):e3003695. doi: 10.1371/journal.pbio.3003695 (PMC13004526; doi:10.1371/journal.pbio.3003695)

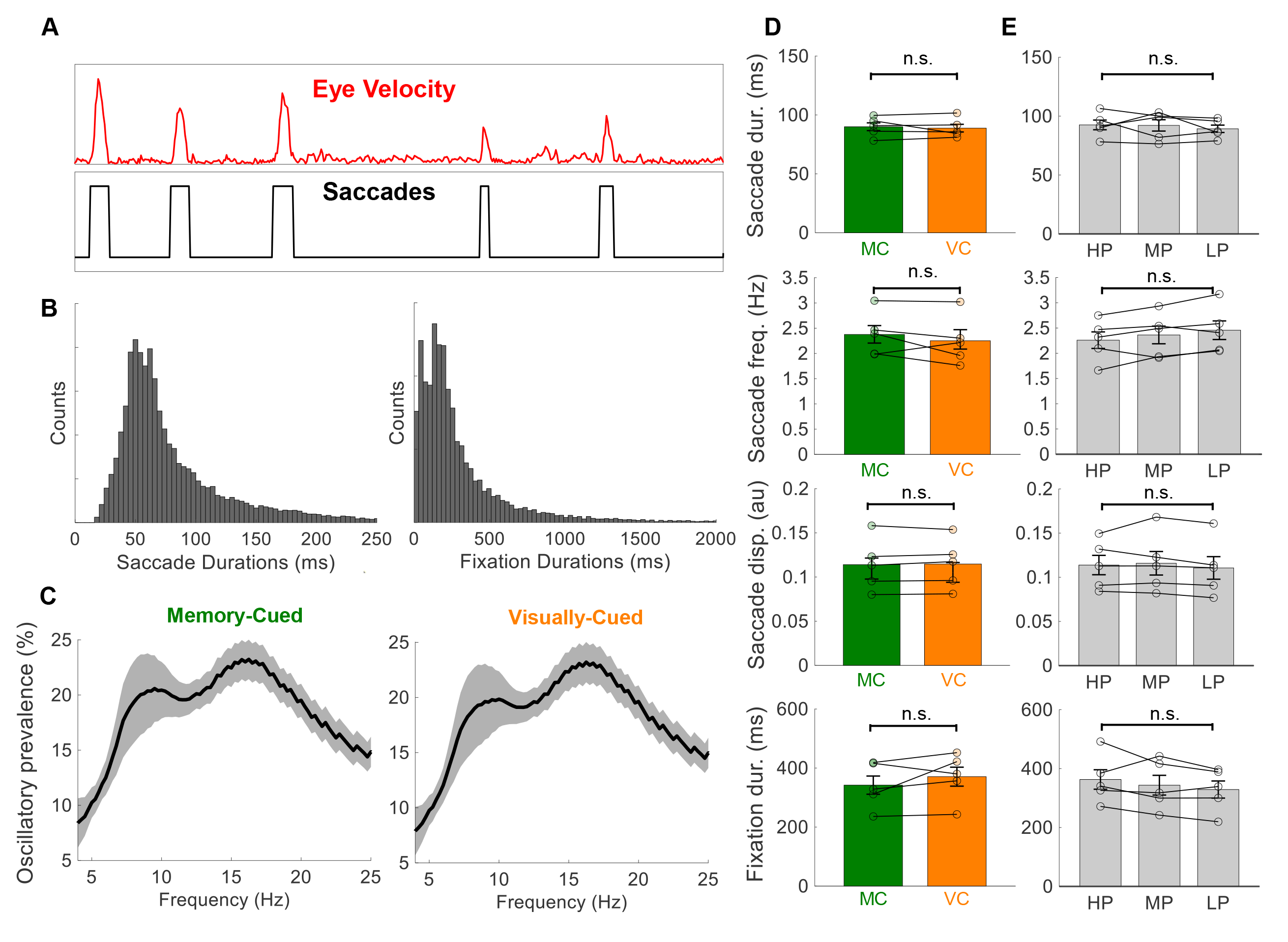

Supplement: S1 Fig — (A) Example two-second segment showing two-dimensional eye velocity (red line) and the corresponding saccade index (black line). (B) Distribution of saccade durations (ms) and fixation durations (ms) across all participants (nparticipants = 5). (C) Prevalence of low-frequency oscillations shown as a percentage (%) of total samples. Oscillatory bouts were detected for individual frequency steps between 4 and 25 Hz during memory-cued and visually-cued navigation. Shaded gray area represents SEM across channels (nchannels = 16). (D) No significant differences in saccade metrics between memory-cued (MC) and visually-cued (VC) conditions: mean duration (p = 0.47), frequency (p = 0.15), displacement (p = 0.31), or fixation duration (p = 0.18). (E) Similarly, saccade metrics did not differ across performance levels. Mean saccade duration, frequency, displacement, and fixation duration did not differ significantly between HP, MP, and LP trials (all p > 0.13). n.s. = not significant. The data underlying this Figure are available here: https://doi.org/10.5281/zenodo.18487389. (S1_Fig.TIF) [file pbio.3003695.s003.tif]

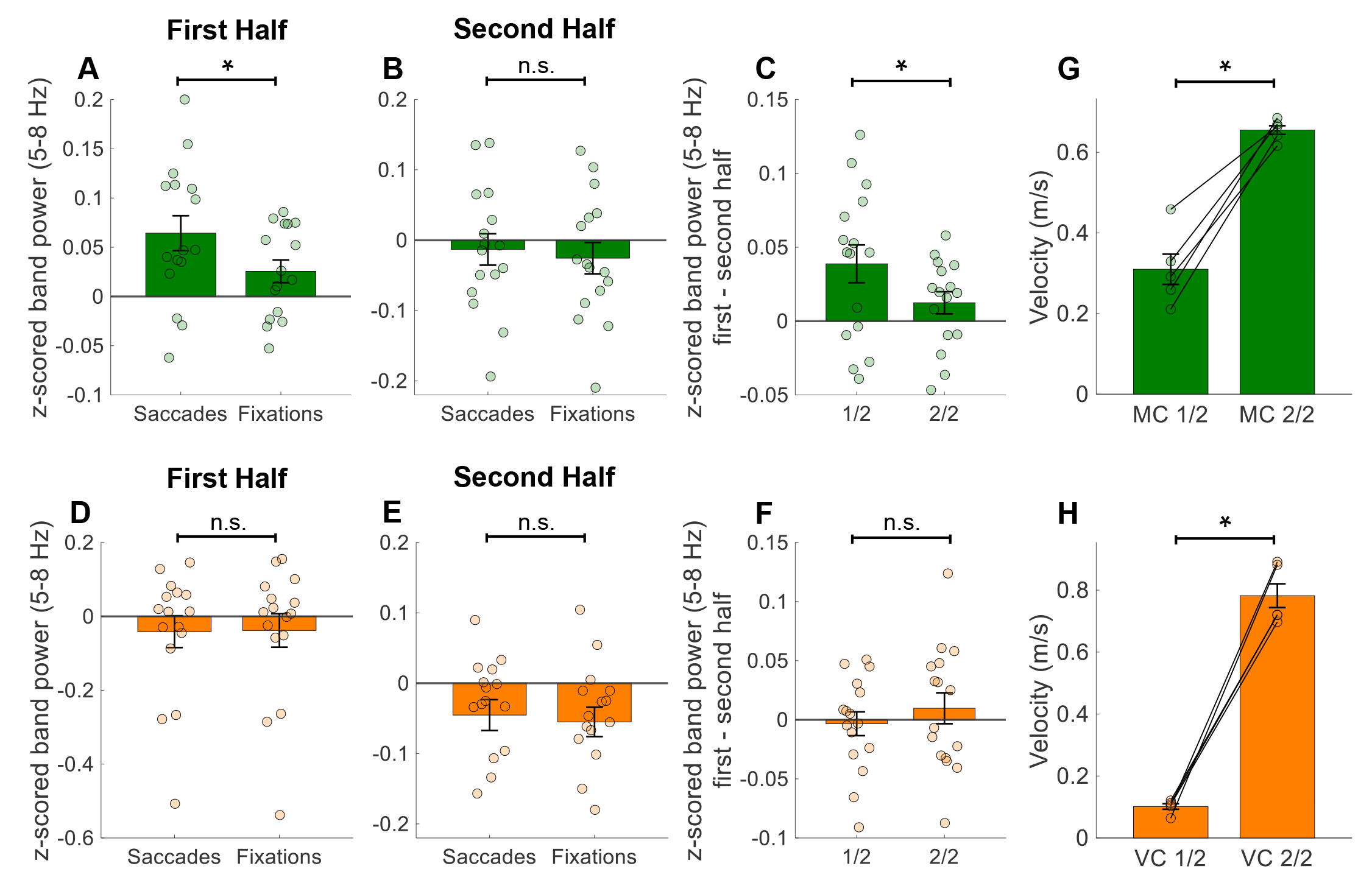

Supplement: S2 Fig — (A–C) For memory-cued (MC) navigation, each trial was divided into equal first-half (planning) and second-half (execution) epochs. Normalized (z-scored) theta (5–8 Hz) activity was significantly higher during saccades compared to fixations in the first half (* p = 0.013; A), but not in the second half (n.s., p = 0.254; B). The difference in saccade-fixation theta modulation between halves was significant (* p = 0.041; C). (D–F) In the visually-cued (VC) navigation condition, no saccade–fixation differences were observed in the first half (n.s., p = 0.546; D), second half (n.s., p = 0.327; E) or their difference (n.s., p = 0.772; F). (G–H) Movement speed increased from planning to execution in both MC navigation (* p = 0.030; G) and visually-cued navigation (* p = 0.031; H), confirming greater locomotor engagement in the latter half of each trial. n.s. = not significant. The data underlying this Figure are available here: https://doi.org/10.5281/zenodo.18487389. (S2_Fig.TIF) [file pbio.3003695.s004.tif]

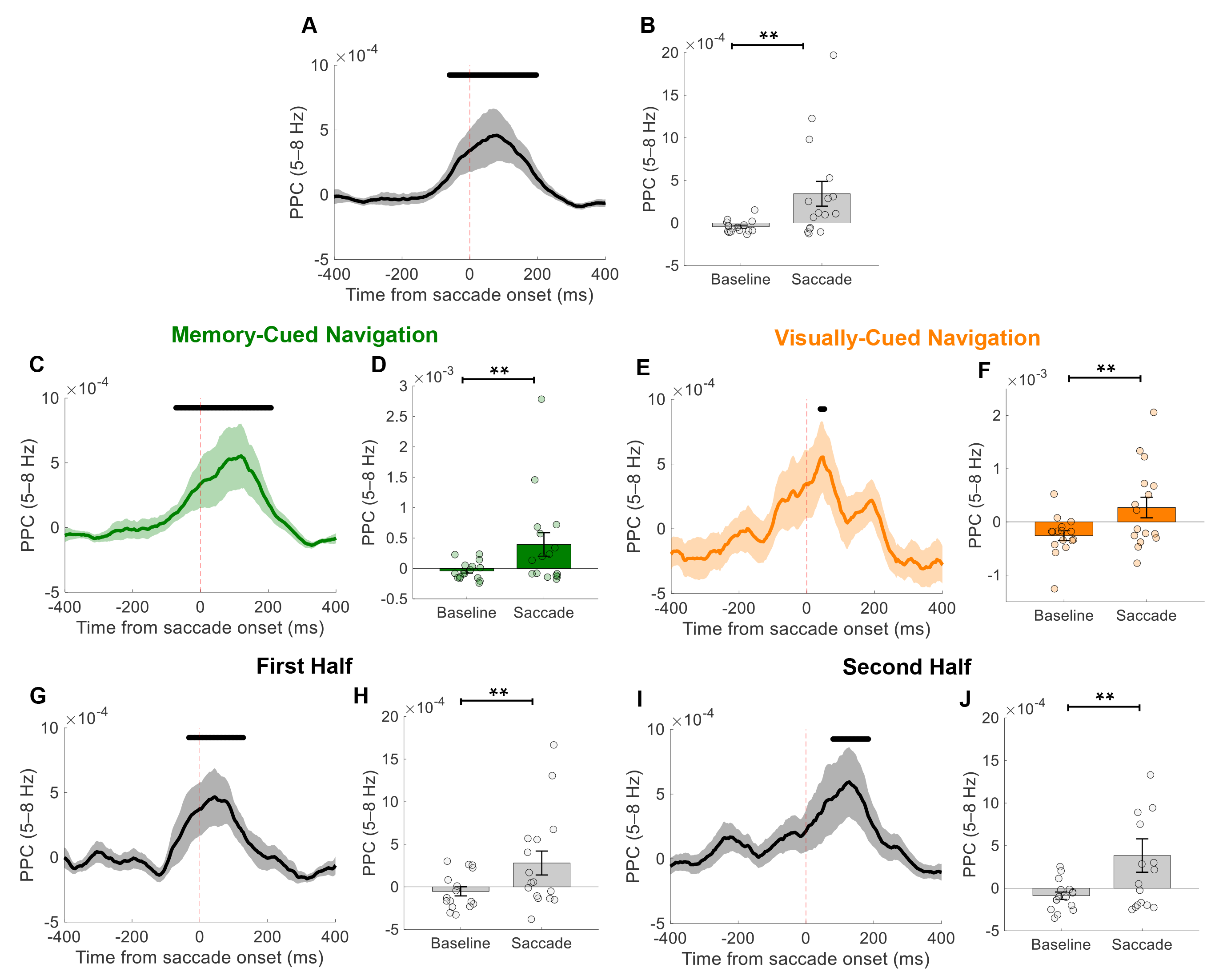

Supplement: S3 Fig — (A–B) Theta phase resetting relative to saccade onset, quantified using pairwise phase consistency (PPC). PPC was significantly elevated from −60 to +196 ms around saccade onset (black bar = p < 0.05, shaded areas represent SEM across participants; A), and PPC during the saccade-onset window (−50 to +200 ms) was significantly higher than an equivalently sized baseline period (−800 to −550 ms, ** p < 0.01; B). (C–F) Time-resolved PPC traces (mean ± SEM across participants, shaded gray) show significant theta phase alignment surrounding saccade onset in both memory-cued (MC) and visually-cued (VC) navigation. (G–J) When trials were divided into early and late halves, significant PPC increases were observed around saccade onset in both periods, indicating consistent phase resetting throughout the trial. Bar plots adjacent to each panel display mean PPC during the saccade-onset window (−50 to +200 ms) and a baseline window (−800 to −550 ms). PPC was significantly higher during the saccade period for all comparisons (** p < 0.01). These results demonstrate that theta phase alignment accompanies saccadic events across both task conditions and throughout the course of navigation. The data underlying this Figure are available here: https://doi.org/10.5281/zenodo.18487389. (S3_Fig.TIF) [file pbio.3003695.s005.tif]

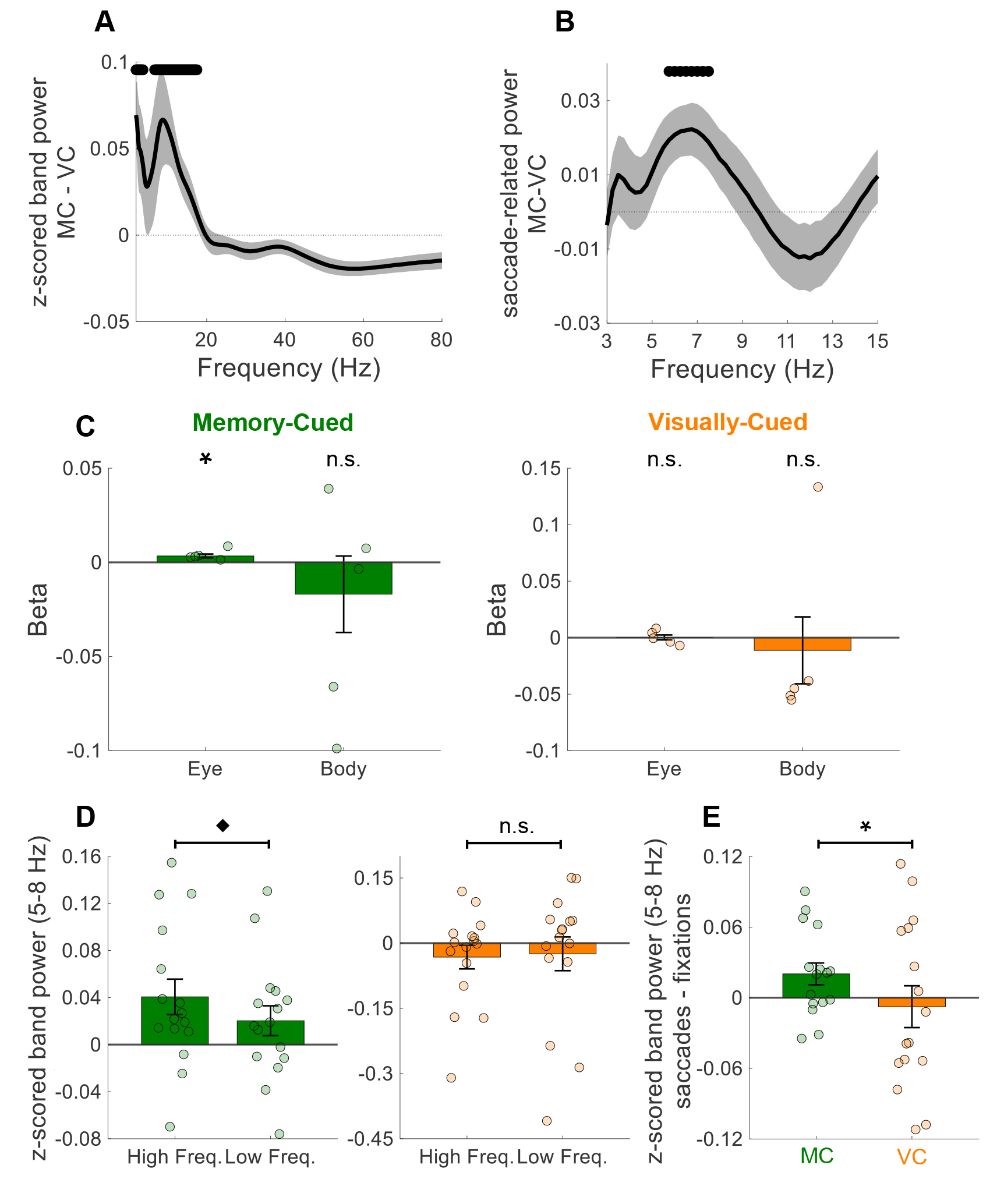

Supplement: S4 Fig — (A) MTL low-frequency power was elevated during memory-cued navigation compared to visually-cued navigation within the 2.0–3.75 Hz and 6.75–17.5 Hz ranges (p < 0.05). (B) The saccade-related oscillatory effect, characterized by an increase in band power during saccades, was more pronounced during memory-cued than during visually-cued navigation, particularly in the theta frequency range (5.75–7.5 Hz). (C) A linear mixed-effects model was employed to assess the concurrent impact of eye and body movement on theta band power, separately for memory- and visually-cued navigation. In memory-cued navigation, eye movement speed significantly contributed to theta power (p = 0.032), whereas body movement speed did not (p = 0.818). In visually-cued navigation, neither eye movement speed (p = 0.436) nor body movement speed (p = 0.656) appeared to modulate changes in theta power. (D) During memory-cued navigation, theta (5–8 Hz) power in the MTL trended higher during high- versus low-saccade frequency periods (p = 0.096). During visually-cued navigation, theta power in the MTL did not significantly differ between high- and low-saccade frequency periods (p = 0.630). (E) Comparison of the high–low entropy theta difference between memory-cued and visually-cued conditions revealed a significant interaction (p = 0.049). For A,B shaded gray = standard error of the mean (SEM) across channels (nchannels = 16). Black horizontal bars = p < 0.05. C shows mean (± SEM) across 5 participants (circles). D,E shows mean (± SEM) across 16 channels (circles). ♦ = p < 0.1, * = p < 0.05, n.s. = not significant. The data underlying this Figure are available here: https://doi.org/10.5281/zenodo.18487389. (S4_Fig.TIF) [file pbio.3003695.s006.tif]

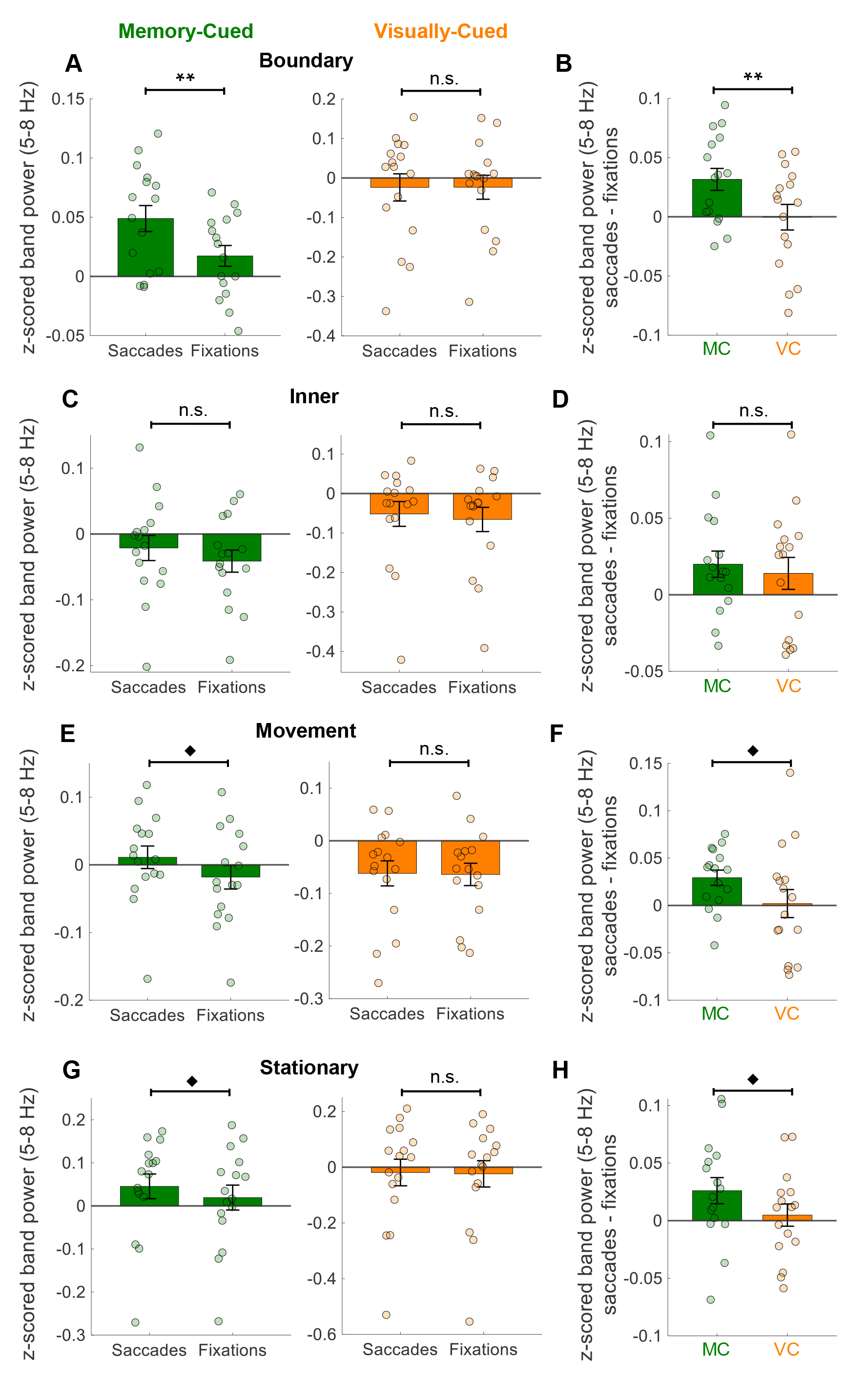

Supplement: S5 Fig — Normalized (z-scored) MTL theta power during saccades is shown separately for memory-cued and visually-cued conditions, in addition to direct comparisons between memory-cued and visually-cued conditions, under four behavioral contexts: (A-B) near boundaries (<1.2 m from the wall), (C-D) inner region (>1.2 m from the wall), (E-F) during movement, and (G-H) while stationary. Saccade-related theta increases were observed primarily during memory-cued navigation, with a significant effect near boundaries and trends in the other contexts. Each point reflects a single MTL channel (N = 16); bars represent mean ± SEM. ♦ = p < 0.1, ** = p < 0.01, n.s. = not significant. The data underlying this Figure are available here: https://doi.org/10.5281/zenodo.18487389. (S5_Fig.TIF) [file pbio.3003695.s007.tif]

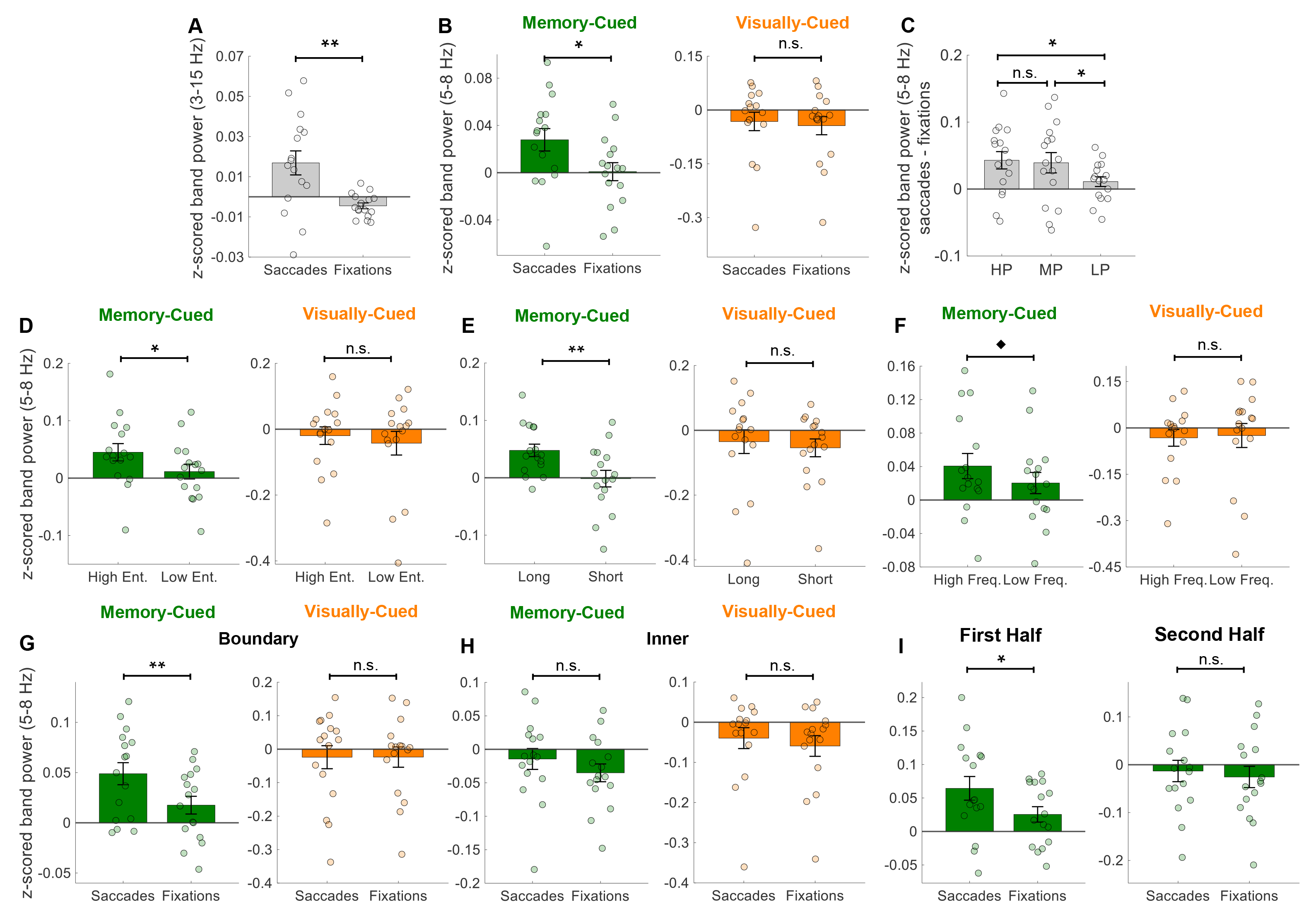

Supplement: S6 Fig — All primary analyses were repeated using BOSC parameters optimized for higher temporal resolution at the expense of frequency resolution (see Materials and Methods). The results remained consistent with the main findings. (A) MTL low-frequency (3–15 Hz) power was significantly higher during saccades than fixations (p = 0.001), replicating Fig 2B. (B) During memory-cued (MC) navigation, MTL theta (5–8 Hz) power increased during saccades relative to fixations (p = 0.013), replicating Fig 3A; no significant theta increase was observed during visually-cued (VC) navigation (p = 0.258), consistent with Fig 3D. (C) Within MC trials, theta power increases were stronger during high-performance (HP; p = 0.012) and medium-performance (MP; p = 0.035) trials compared to low-performance (LP) trials, consistent with Fig 3H. (D) Theta power was higher during high- versus low-entropy periods in MC navigation (p = 0.023) but not during VC navigation (p = 0.158), replicating Fig 4E and 4F. (E) Theta power was higher during long versus short saccades in MC navigation (p = 0.004) but not during VC navigation (p = 0.205), replicating Fig 4B. (F) Theta power showed a trend toward being higher during high- versus low-frequency saccades in MC navigation (p = 0.096) but not during VC navigation (p = 0.630), replicating S4D Fig. (G) Theta power was higher during saccades compared to fixations in boundary regions during MC navigation (p = 0.007) but not during VC navigation (p = 0.506), replicating S5A Fig. (H) Theta power did not differ between saccades and fixations in inner-room regions for either MC or VC navigation (MC, p = 0.130; VC, p = 0.164), replicating S5C Fig. (I) Theta power was higher during saccades compared to fixations during the first half of MC trials (p = 0.013) but not during the second half (p = 0.251), replicating S2A and S2B Fig. All panels show mean ± SEM across 16 MTL channels (circles). * p < 0.05, ** p < 0.01, ♦ p < 0.1; n.s. = not significant. The data underlying t [file pbio.3003695.s008.tif]
