# Supplementary material for: Eye movements reflect memory-related theta activity in the human brain
Source: PLoS Biol. 2026 Mar 16;24(3):e3003695. doi: 10.1371/journal.pbio.3003695 (PMC13004526; doi:10.1371/journal.pbio.3003695)
Supplement: Attachment — Submitted filename: Response_to_Reviewers.pdf [file pbio.3003695.s012.pdf]

Response to Reviewers

Dear Reviewers,

We sincerely thank you for your thorough and constructive reviews of our manuscript, *Eye Movements Reveal Memory-Related Theta Activity in the Human Brain*. We greatly appreciate the thoughtful feedback, which has helped us clarify and strengthen the manuscript. Below, we provide detailed responses to all comments. Text added or revised in the manuscript is indicated in blue text both below and in the annotated manuscript.

Reviewer #1: *This manuscript presents an elegant and technically sophisticated investigation into the relationship between saccadic eye movements and medial temporal lobe (MTL) theta activity during ambulatory spatial navigation in humans. Using mobile intracranial EEG recordings from five patients with implanted RNS devices, combined with high-resolution motion tracking and eye-tracking, the authors demonstrate that theta power increases during saccades—but selectively so under memory-guided conditions. Moreover, these saccade-related theta increases are most pronounced during trials with better memory performance, suggesting a functional link between visual sampling and memory success. This study offers a compelling framework to reconcile interspecies differences in theta dynamics by highlighting how humans use vision-based active sensing (saccades) in ways that may parallel whisking in rodents. The authors' interpretations are well-argued and thoughtfully positioned within the existing literature. The analytical rigor, creative use of mobile iEEG technology, and translational relevance of the findings make this a valuable contribution. Below, I outline the major strengths, followed by two substantive suggestions for improvement and several minor comments.*

Major Strengths
1. Innovative Methodology: The authors leverage state-of-the-art mobile iEEG to simultaneously record hippocampal activity, body movement, and eye tracking in freely moving humans—an impressive technical achievement that allows for ecologically valid cognitive neuroscience.
2. Cognitive Specificity: By contrasting memory-cued with visually guided navigation and matching oculomotor metrics across these conditions, the study isolates the cognitive context (memory demand) as the key modulator of saccade-related theta increases.
3. Behavioral Relevance: The link between theta power and successful memory trials (based on detour distance) provides meaningful evidence that theta is not merely movement- or attention-related but behaviorally significant.
4. Cross-Species Integration: The discussion thoughtfully relates the findings to species differences in active sensing, offering a unifying framework for understanding theta across rodents, non-human primates, and humans.
5. Analytical Rigor: The authors apply robust and transparent statistical methods—including leave-one-subject-out validation, permutation testing, and sensitivity checks across time-frequency resolutions—demonstrating excellent methodological care.

Response: We thank Reviewer #1 for the exceptionally positive assessment of our work and for recognizing the innovation, analytical rigor, and theoretical contribution of our study. We appreciate the reviewer's insightful suggestions, which prompted additional analyses addressing each point below and further strengthened the interpretation of our findings.

Major Suggestions for Improvement

1. Disentangling Planning from Memory Retrieval: While the authors interpret saccade-theta coupling as a signature of memory engagement, it is equally plausible that these effects reflect visuospatial planning demands, which are inherent in the memory-cued condition. Participants must retrieve the target location but also plan a route to an unseen goal, engaging additional cognitive operations distinct from retrieval per se.

To strengthen this interpretation, the authors could consider:

Comment 1 [C1]: *Segmenting trials into pre-movement (planning) and in-motion (execution) epochs to compare theta modulation across these phases.*

Response: We thank the reviewer for this thoughtful suggestion. To address it, we performed an additional analysis in which we divided each memory-cued (MC) trial into two equal halves, corresponding to planning (first half) and execution (second half) phases. Theta power was significantly higher during saccades compared to fixations in the **first half** ($N_{channels} = 16$, $t = 2.29$, $p = 0.013$; S2A Fig) but not in the **second half**

($N_{channels}$ = 16, t = 0.68, p = 0.254; S2B Fig). Importantly, the **difference in saccade–fixation theta modulation between the first and second halves** was significant ($N_{channels}$ = 16, t = 1.79, p = 0.041; Fig. S2C). In addition to this half-trial analysis, we also examined pre-movement (planning) and in-motion (execution) epochs defined independently of trial halving. These showed a similar pattern, significant saccade-related theta increases during planning ($p < 0.05$) but not execution (p = n.s.). These findings suggest that saccade-related theta coupling is strongest during the early, planning-dominant portion of the trial, indicating that theta dynamics could reflect both memory retrieval and planning processes. For completeness, we repeated the same half-trial analysis for the visually-cued (VC) navigation condition. As expected, the results showed no significant saccade–fixation modulation: first half ($N_{channels}$ = 16, t = –0.120, p = 0.546; S2D Fig), second half ($N_{channels}$ = 16, t = 0.459, p = 0.327; S2E Fig), and the difference between halves ($N_{channels}$ = 16, t = –0.761, p = 0.772; S2F Fig). Finally, we confirmed that movement speed increased from planning to execution, consistent with behavioral expectations. Velocity was significantly higher in the second half than the first half for both MC navigation ($N_{participants}$ = 5, t = –8.400, p = 0.030; S2G Fig) and visually-cued navigation ($N_{participants}$ = 5, t = –13.513, p = 0.031; S2H Fig). We have added these analyses and corresponding plots to the revised manuscript (*Results*, "Saccade-Related Theta Activity is Strongest During Successful and Early Phases of Memory-Guided Navigation", S2 Fig) and also updated our abstract and discussions sections accordingly:

Results (pg. 9, lines 219-236):

"To further examine the temporal dynamics of theta activity within memory-guided navigation, we divided each memory-cued trial into an early ("planning") and late ("execution") half. The first half corresponded to initial route planning and search behavior, while the second half reflected execution of the recalled path toward the hidden target. Theta power during saccades was significantly higher than during fixations in the first half ($N_{channels}$ = 16, t = 2.29, p = 0.013; S2A Fig), but not in the second half ($N_{channels}$ = 16, t = 0.68, p = 0.254; S2B Fig). The difference in saccade–fixation theta modulation between halves was significant ($N_{channels}$ = 16, t = 1.79, p = 0.041; S2C Fig). In contrast, visually-cued navigation showed no significant saccade-fixation modulation in either half: first half ($N_{channels}$ = 16, t = –0.120, p = 0.546; S2D Fig), second half ($N_{channels}$ = 16, t = 0.459, p = 0.327; S2E Fig), or their difference ($N_{channels}$ = 16, t = –0.761, p = 0.772; S2F Fig). We also confirmed that movement speed increased from planning to execution, consistent with behavioral expectations. Velocity was significantly higher in the second half than the first half for both memory-cued navigation ($N_{participants}$ = 5, t = –8.400, p = 0.030; S2G Fig) and visually-cued navigation ($N_{participants}$ = 5, t = –13.513, p = 0.031; S2H Fig), indicating that this temporal modulation of theta is not explained by changes in movement alone. Together, these findings show that saccade-related MTL theta activity is strongest during the early, planning-dominant portion of memory-guided trials and during successful retrieval, supporting a role for theta in anticipatory visual search and memory-driven planning rather than low-level motor execution."

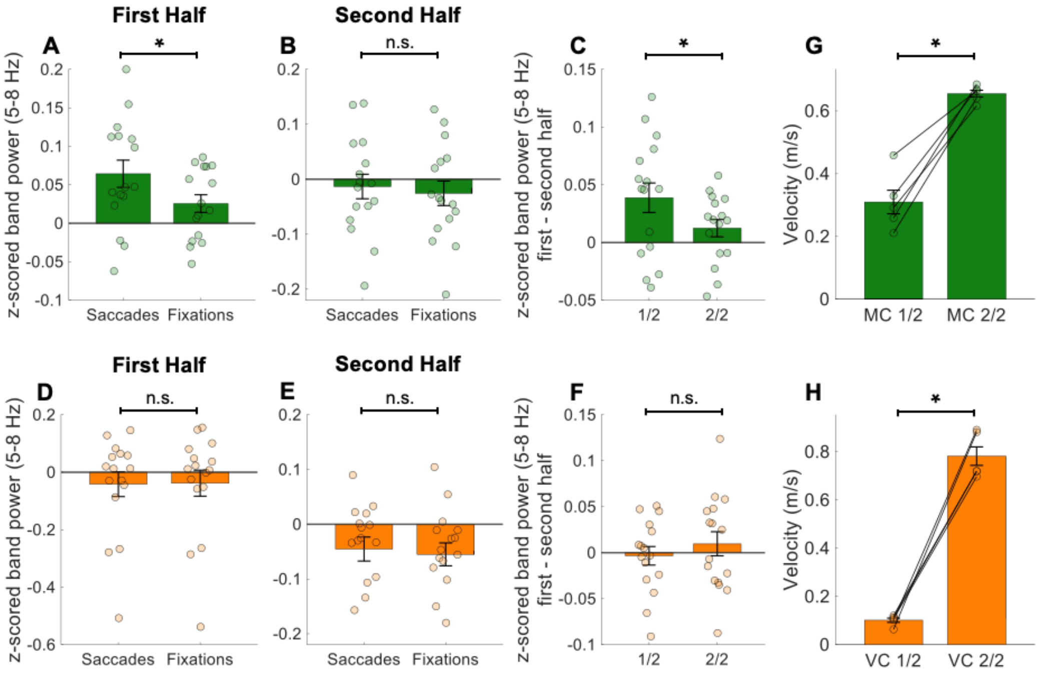

S2 Fig. Saccade-related theta modulation differs between planning and execution phases of memory-cued trials. (A-C) For memory-cued (MC) navigation, each trial was divided into equal first-half (planning) and second-half (execution) epochs. Normalized (z-scored) theta (5-8 Hz) activity was significantly higher during saccades compared to fixations in the first half (* p = 0.013; **A**), but not in the second half (n.s., p = 0.254; **B**). The difference in saccade-fixation theta modulation between halves was significant (* p = 0.041; **C**). (**D–F**) In the visually-cued (VC) navigation condition, no saccade–fixation differences were observed in the first half (n.s., p = 0.546; **D**), second half (n.s., p = 0.327; **E**) or their difference (n.s., p = 0.772; **F**). (**G–H**) Movement speed increased from planning to execution in both MC navigation (* p = 0.030; **G**) and visually-cued navigation (* p = 0.031; **H**), confirming greater locomotor engagement in the latter half of each trial. n.s. = not significant.

C2: *Categorizing saccades as goal-congruent or non-congruent, and testing whether theta is more strongly modulated by those aligned with future path trajectories.*

Response: Thank you for this helpful suggestion. As requested, we categorized saccades based on whether they moved closer to the upcoming goal location (convergent) or farther from it (divergent) and compared theta modulation between these groups. Although the mean values were numerically higher for goal-convergent saccades, the difference was not statistically reliable ($N_{channels}$ = 16, t = 0.586, p = 0.273, Fig. R1 below). Although we do not incorporate this analysis into the manuscript, we believe this remains an interesting question that future studies with larger datasets should explore further.

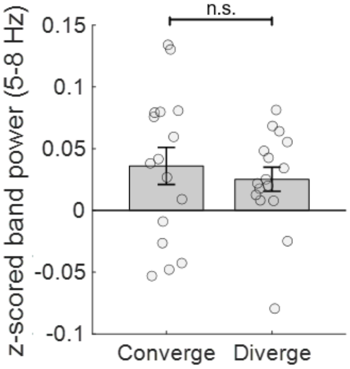

Figure R1. Theta modulation during goal-convergent vs. goal-divergent saccades. Saccades were classified based on whether they moved gaze closer to (convergent) or farther from (divergent) the upcoming goal. Although convergent saccades showed slightly higher mean theta activity, the effect was not significant across channels ($N_{channels}$ = 16, t = 0.586, p = 0.273).

C3: *Computing scanpath entropy or dispersion metrics to quantify planning load, and examining how they relate to theta power.*

Response: We thank the reviewer for this suggestion. To examine the relationship between eye-movement complexity and theta activity, we computed **scanpath entropy** using a grid-binned spatial occupancy approach (Melnyk et al., 2024; Harris et al., 1986). The visual field was divided into 30 × 30 bins, and Shannon entropy was calculated for each data point within 1-second sliding windows. For each participant, we then performed a median split of entropy values into high- and low-entropy time points and compared the corresponding z-scored theta power (5–8 Hz). In memory-cued navigation, theta power was significantly higher during high-entropy than low-entropy periods ($N_{channels}$ = 16, t = 2.06, p = 0.023; Figure 4E), whereas in visually-cued navigation the difference was not significant ($N_{channels}$ = 16, t = 1.03, p = 0.158; Figure 4F). These results suggests that **greater gaze dispersion or more exploratory scanpaths are associated with stronger theta activity, particularly during memory-guided navigation**. We have added these analyses and corresponding plots to the revised manuscript (*Results*, "Scanpath entropy" in *MTL Theta Activity Reflects Exploratory Eye Movements During Memory-Guided Navigation* section; pg. 11; lines 275-287; Figure 4):

"To quantify the spatial complexity of eye movements, we computed scanpath entropy using a grid-binned spatial occupancy approach [21,22]. The visual field was divided into 30 × 30 bins, and Shannon entropy was calculated within 1-second sliding windows. For each participant, we then performed a median split of entropy values into high- and low-entropy time points and compared the corresponding 5–8 Hz z-scored theta power. In memory-cued navigation, theta power was significantly higher during high-entropy than low-entropy periods ($N_{channels}$ = 16, t = 2.06, p = 0.023; Figure 4E), whereas in visually-cued navigation the difference was not significant ($N_{channels}$ = 16, t = 1.03, p = 0.158; Figure 4F). These results suggests that greater gaze dispersion or more exploratory scanpaths are associated with stronger theta activity, particularly during memory-guided navigation. Together, these results indicate that MTL theta power may track multiple aspects of visual exploration, including saccade magnitude, frequency, and spatial dispersion, during memory-guided

navigation. Theta thus appears to reflect internal cognitive processes linked to active search and spatial planning rather than simple oculomotor behavior."

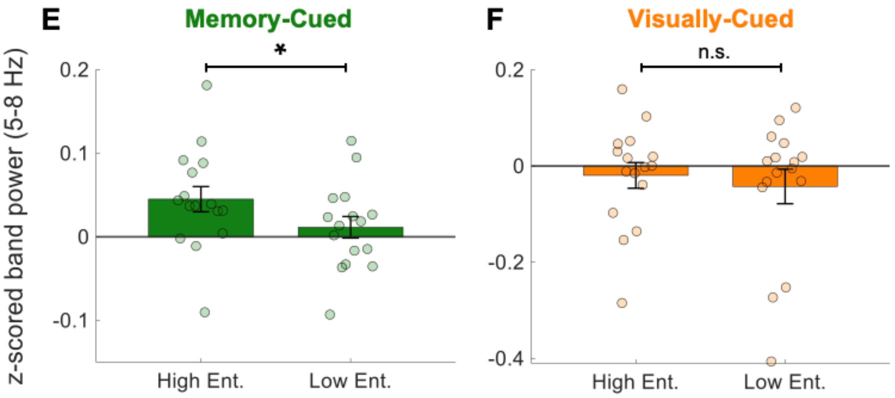

Figure 4. (E,F) Mean normalized (z-scored) 5–8 Hz theta power during high- and low-entropy periods for each condition. Theta power was significantly higher during high-entropy periods in MC navigation (* $p = 0.023$; **E**) and not significant in VC navigation (n.s., $p = 0.158$; **F**).

C4: *If theta increases are stronger during structured, anticipatory eye movement patterns or early trial epochs, this would support a broader role for theta in prospective visual planning, beyond memory recall alone.*

Response: We appreciate this comment. Consistent with this suggestion, our new analyses support a broader role of theta in anticipatory planning processes. Specifically, when memory-cued trials were divided into early (planning) and late (execution) halves, theta power during saccades relative to fixations was significantly greater in the first half compared to the second half, with a significant difference between halves (S2 Fig). Furthermore, our scanpath entropy analysis revealed that periods of higher gaze dispersion, reflecting greater exploratory or planning load, were associated with increased theta power during memory-guided navigation (Figure 4E-F). Together, these findings indicate that theta dynamics are strongest during early, structured, and exploratory viewing periods, suggesting a role in prospective visual planning in addition to memory retrieval. We have incorporated these results and interpretation into the revised *Abstract* and *Discussion* sections:

Discussion (pg. 15, lines 379-388):

"Theta activity was not uniform across the course of a trial. Saccade-related theta power increases were stronger during the early, planning-dominant phase of memory-cued navigation than during later execution. Moreover, periods of higher scanpath entropy, reflecting more exploratory and dispersed gaze behavior, were associated with greater theta power. The link between exploratory viewing and theta echoes prior work showing that hippocampal theta in both humans and monkeys increases during active visual search [2,38]. This pattern suggests that theta may help organize internal predictions or spatial hypotheses before movement, supporting a transition from memory recall to forward-looking planning. In this view, hippocampal theta could act as a bridge between retrieving past experiences and simulating future trajectories, linking perception, attention, and memory into a cohesive planning process."

Abstract (pg. 2, lines 44-47):

"Together, these findings reveal that memory-related theta dynamics in the human MTL are tightly coupled to exploratory visual search and prospective planning during memory-guided navigation, revealing a mechanism by which saccades may help organize mnemonic computations in naturalistic settings."

2. Theta Phase Resetting and Temporal Alignment: The manuscript focuses on theta amplitude changes, but prior studies in both humans and non-human primates suggest that saccades can induce phase resetting in MTL theta. Such phase alignment may temporally organize neural ensembles for memory encoding and retrieval, and could be present even in the absence of large amplitude changes. I encourage the authors to explore:

C5: *Whether theta phase aligns to saccade onset, using measures like inter-trial phase coherence or pairwise phase consistency.*

Response: We thank the reviewer for this valuable suggestion to complement our amplitude analyses with a phase-based measure. To examine whether theta activity exhibits phase alignment to saccade onset, we

computed **pairwise phase consistency (PPC)**, which quantifies phase similarity across saccades and is unbiased by trial count (Vinck et al., 2010; Hoffman et al., 2013). We observed clear evidence of **theta phase resetting** around saccade onset, with PPC significantly elevated from –60 to +196 ms relative to saccade onset. To quantify this effect further, we compared PPC during a defined saccade-onset period (–50 to +200 ms) with a baseline period of equal duration (–800 to –550 ms), following prior work (Hoffman et al., 2013). PPC was significantly higher during the saccade-onset period ($p < 0.001$). These findings demonstrate that saccades induce reliable theta phase resetting in medial temporal lobe recordings, consistent with phase-resetting mechanisms proposed to temporally organize neural activity during perception and memory processing. We have added this new analysis and figure to the revised manuscript (*Results*, "Saccades Induce Theta Phase Resetting Independent of Memory and Trial Phase"; S3 Fig):

Results (pg. 12, lines 290-297):

"To test whether saccades elicit phase resetting of medial temporal lobe (MTL) theta, we computed pairwise phase consistency (PPC) across saccades [23]. Consistent with prior studies in humans and non-human primates demonstrating saccade-locked theta phase resetting [2,6,24,25], PPC was significantly elevated around saccade onset from –60 to +196 ms relative to saccade onset ($N_{channels} = 16$, black bar = $p < 0.05$; S3A Fig). Using a predefined saccade-onset window (–50 to +200 ms) and a baseline period of equal duration (–800 to –550 ms) [6], we confirmed that PPC during saccades was significantly higher than baseline across all navigation conditions ($N_{channels} = 16$, $t = 2.62$, $p = 0.002$; S3B Fig), indicating reliable theta phase resetting associated with saccadic events."

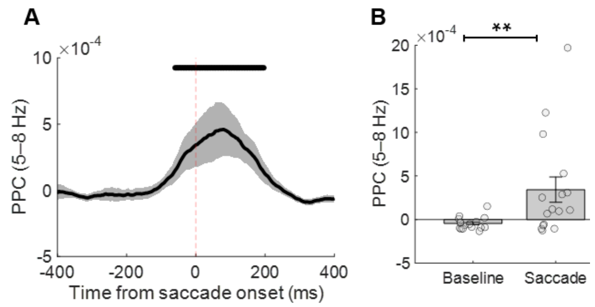

S3 Fig. Theta phase resetting to saccade onset. (A–B) Theta phase resetting relative to saccade onset, quantified using pairwise phase consistency (PPC). PPC was significantly elevated from –60 to +196 ms around saccade onset (black bar = $p < 0.05$, shaded areas represent SEM across participants; **A**), and PPC during the saccade-onset window (–50 to +200 ms) was significantly higher than an equivalently sized baseline period (–800 to –550 ms, ** $p < 0.01$; **B**).

C6: *Whether phase resetting varies by task condition or navigation phase (e.g., stronger near goal locations or early in trials).*

Response: We examined whether theta phase alignment varied across both task conditions and different parts of the trial. Significant phase resetting around saccade onset was observed in both memory-cued and visually-cued navigation conditions (S3C-F Fig). To test for variation over time, each trial was divided into early and late halves corresponding to the initial and later parts of the trial (as defined for the power analysis). Significant saccade-related phase resetting was present in both halves ($t = 2.51$, $p = 0.005$, early; $t = 2.31$, $p = 0.004$, late; S3G-J Fig), indicating that theta phase alignment accompanies saccadic events across both MC and VC navigation and throughout the course of the trial. These findings suggest that saccade-related theta phase resetting may reflect a general organizing mechanism that operates across task contexts and temporal epochs of navigation. We have added these results to the revised manuscript (*Results*, "Saccades Induce Theta Phase Resetting Independent of Memory and Trial Phase"; S3 Fig):

Results (pg. 12, lines 298-315):

"When analyzed separately by task condition, phase resetting occurred in both memory-cued and visually-cued navigation. In memory-cued trials, PPC increased from –72 to +208 ms ($N_{channels} = 16$, black bar = $p < 0.05$; S3C Fig) and was significantly higher during saccades than baseline ($N_{channels} = 16$, $t = 2.12$, $p = 0.006$; S3D Fig). In visually-cued trials, a shorter window of significant PPC was observed from +40 to +52 ms ($N_{channels} = 16$, black bar = $p < 0.05$; S3E Fig) and PPC during saccades was also significantly higher than baseline ($N_{channels} = 16$, $t = 2.70$, $p = 0.005$; S3F Fig). The presence of phase resetting in both memory-cued and visually-cued navigation suggests that saccade-linked theta synchronization is not exclusively memory-dependent but may instead reflect a general mechanism coupling visual sampling with MTL timing. The longer post-saccade duration in the memory-cued condition may point to sustained mnemonic or planning-related processes extending beyond immediate sensory updating.

To assess whether phase resetting varied across trial phases, we divided each trial into early ("planning") and late ("execution") halves. Theta phase resetting was significant in both early ($N_{channels}$ = 16, t = 2.51, p = 0.005) and late ($N_{channels}$ = 16, t = 2.31, p = 0.004) halves (S3G-J Fig), suggesting that this synchronization accompanies saccadic events throughout navigation. Together, these findings suggest that saccades serve as temporal anchors for theta phase resetting in the human MTL, supporting both visual exploration and higher-order cognitive operations such as planning and memory retrieval."

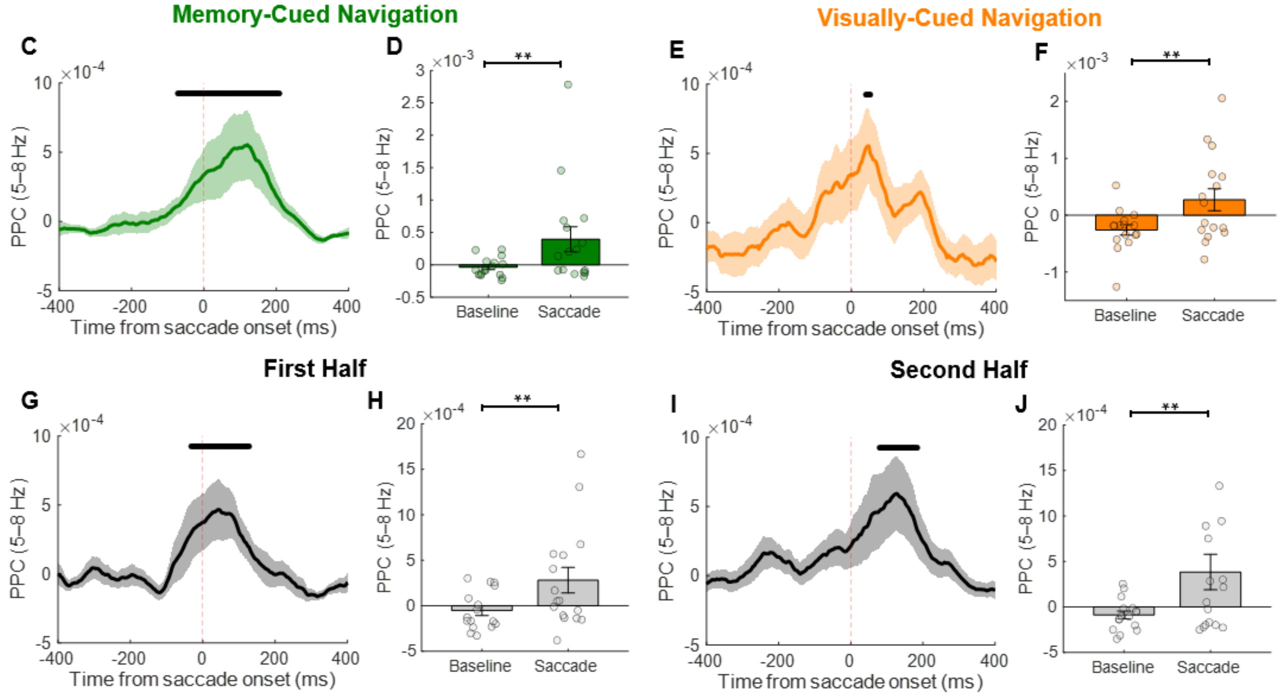

S3 Fig. Theta phase resetting across task conditions and trial epochs. Pairwise phase consistency (PPC) was used to quantify theta phase resetting relative to saccade onset across task conditions and trial epochs. **(C–F)** Time-resolved PPC traces (mean ± SEM across participants, shaded gray) show significant theta phase alignment surrounding saccade onset in both memory-cued (MC) and visually-cued (VC) navigation. **(G–J)** When trials were divided into early and late halves, significant PPC increases were observed around saccade onset in both periods, indicating consistent phase resetting throughout the trial. Bar plots adjacent to each panel display mean PPC during the saccade-onset window (–50 to +200 ms) and a baseline window (–800 to –550 ms). PPC was significantly higher during the saccade period for all comparisons (** $p < 0.01$). These results demonstrate that theta phase alignment accompanies saccadic events across both task conditions and throughout the course of navigation.

C7: *Whether trial-by-trial phase consistency predicts behavioral performance (e.g., detour error), thereby complementing the amplitude-based findings. Such analyses could provide a more mechanistic account of how saccades organize memory-relevant computations in the MTL.*

Response: We agree that examining whether trial-by-trial phase consistency predicts behavioral performance would provide a useful complement to the amplitude-based results. To address this, we computed Pairwise Phase Consistency (PPC) across saccade onsets within each trial for each channel and correlated these PPC values with trial-level performance (detour error). This analysis did not reveal a significant relationship (p = 0.553), indicating that trial-by-trial variations in theta phase alignment did not predict performance in our dataset. This null effect is consistent with our observation that PPC increases occurred during both memory-guided and visually-cued navigation, suggesting that saccade-related phase resetting reflects a general visuomotor alignment process rather than a mechanism specifically tied to memory performance. Nonetheless, the question of whether phase alignment contributes to memory-relevant computations remains important, and future studies using complementary tasks or analyses will be useful for further addressing this possibility.

Reviewer #2: *The authors examine the relationship between eye movements, ambulatory motion, and MTL theta power in intracranial EEG recordings from five freely moving patients performing a spatial navigation task. They find that MTL theta power is increased during saccades, specifically during memory guided navigation, that this correlates with navigational accuracy, and is greater for longer saccade distances. These are rare data, the results are novel and should be of interest to the wider neuroscience community. I just have a few concerns about the statistical analyses presented here, and require clarification on a few aspects of the empirical methods.*

Response: We thank Reviewer #2 for the thoughtful and constructive review and for recognizing the novelty and significance of these rare intracranial recordings during freely moving navigation. We appreciate the helpful comments regarding the statistical analyses and methodological details. In the revised manuscript, we have clarified these procedures and added complementary analyses addressing each point below, which further strengthen the rigor and interpretation of our findings.

Major Comments

C1: *It is not clear to me - from either the main text, Figure 1 caption, or Methods - how participants learned the invisible target locations?*

Response: We thank the reviewer for noting this point. Participants were not explicitly shown the hidden target locations before the task. Instead, participants were instructed to freely explore the environment during the initial trials of the first task block, until they located each invisible target (0.7 m diameter). Each time a target was reached, an auditory cue signaled success, allowing participants to gradually learn and remember the target locations through experience. We have clarified this procedure in the revised *Methods* section and Figure 1 caption:

Figure 1 caption (pg. 5, lines 105-110):

"The environment contained twenty visible wall-mounted signs and three invisible circular target locations (0.7 m diameter). At the start of the task, participants freely explored the room to locate the invisible targets; each time a target was reached, an auditory tone signaled success, allowing them to gradually learn and remember these locations through experience. The task then alternated between two conditions: "visually-cued" navigation, during which participants navigated to a wall-mounted sign (e.g., "Blue 3"), and "memory-cued" navigation, during which they recalled and navigated to the previously learned invisible targets (e.g., "T")."

Methods (pg. 19, lines 473-486):

"Participants performed a memory-guided spatial navigation task while freely moving within a rectangular room (approximately 5.9 × 5.2 m; ~19.4 × 17.1 ft). Twenty wall-mounted signs displaying color-number pairs served as navigational cues, and three predefined circular target locations (0.7 m diameter) were invisibly positioned within the space (Figure 1C). Trials alternated between two types: (1) visually-cued navigation, where participants walked to a visible wall sign (e.g., "yellow 4"); and (2) memory-cued navigation, where they searched for a previously learned but currently hidden target location (e.g., "T"). Hidden targets were not shown prior to the task. Instead, during initial trials, participants were instructed to explore until they reached the correct location, at which point an audio tone signaled that they were within the target's 0.7 m radius. This auditory feedback was repeated each time the target was reached, strengthening memory across trials. Each participant completed multiple blocks of alternating trial types (2–4 blocks, ~15 min each). The same hidden targets were repeated within a block (four for Participant 1; three for Participants 2–5), and the order of targets varied across blocks. During memory-cued trials, participants could either search freely or walk directly to the hidden target if recalled."

C2: *At various junctures, the authors present statistical results across channels alongside results across patients. This implicitly assumes that data from each iEEG electrode contact can be treated as an independent observation, which is clearly not the case. As such, all statistical analyses across channels should be removed from the manuscript. As far as I can tell, this will not affect the overall pattern results at all, as the across channel / across patient statistical results are always consistent.*

Response: We thank the reviewer for this important comment and agree that electrode contacts within a subject cannot be treated as independent observations. To address this, we revised our statistical approach to use the **Permutation Analysis of Linear Models (PALM) framework** (Winkler et al., 2014), which constrains permutations within subjects and thereby limits exchangeability to the individual participant level. This approach maintains appropriate statistical independence while preserving sensitivity to detect true effects and providing high-resolution p-values. Accordingly, all "channel-level" statistical results throughout the manuscript have been reanalyzed using the PALM method, and the corresponding text, figures, and tables have been updated throughout the manuscript. The overall pattern of results remains consistent with the original findings but is now supported by a more rigorous statistical framework. We have added this revision to the *Methods* section:

Methods (pg. 24, lines 620-623):

"All channel-level analyses were implemented using the PALM framework [17], which constrains permutation exchangeability within each participant, allowing valid inference without assuming independence across electrodes."

C3: *On a related note, please report the values of test statistics and degrees of freedom (or simply, n=5) for all statistical tests throughout the manuscript. In places (e.g. when examining whether 'differences in saccadic modulation of theta power could be explained by variations in eye movement behavior across conditions') this information is not given, and so it is not possible to ascertain whether these analyses are across channels or across participants. It should also be made clear in the main text whether test results are from one- or two-sided tests.*

Response: We thank the reviewer for this helpful suggestion to improve clarity and transparency. In the revised manuscript, we now report all relevant test statistics and sample sizes throughout the Results section. For channel-level analyses, we specify the number of channels ($N_{channels}$ = 16) and note that these tests were conducted using the PALM framework, which constrains permutations within participants to ensure appropriate statistical independence. We also explicitly indicate for each analysis whether inference was performed at the channel level or the participant level. In addition, we now state in the main text that all statistical tests were one-sided, consistent with our directional hypotheses that theta power would be greater during saccades than fixations and stronger during memory-cued than visually-cued navigation. We also corrected the permutation-based p-value calculation to include permutations with values greater than or equal to the observed test statistic (i.e., including ties), as required for accurate estimation. This refinement led to small increases in some p-values but did not change any statistical conclusions. Together, these revisions ensure that the statistical reporting is complete, transparent, and fully interpretable for readers evaluating the evidence supporting each result.

Statistical Testing (as included in Methods pg. 24, lines 610-624):

"For participant-level analyses (e.g., S1D Fig), statistical comparisons were performed using one-sided permutation tests with 10,000 permutations, as all hypotheses were directional (e.g., expecting higher theta power during saccades vs. fixations, or during memory-cued vs. visually-cued navigation). For paired data, condition labels were shuffled, and the mean difference was recalculated repeatedly to build a null distribution. The observed mean difference was then tested against this distribution. To assess whether a single set of values (e.g., mean power) differed from zero, we randomly flipped the sign of each value, recalculated the mean, and repeated this 10,000 times to create a null distribution. The true mean was then compared to this distribution. In all permutation-based tests, p-values were computed by counting permutations with values greater than or equal to the observed statistic (i.e., including ties). This approach provides an accurate estimate of the probability of observing the test statistic under the null hypothesis and prevents underestimation of p-values. All channel-level analyses were implemented using the PALM framework [17], which constrains permutation exchangeability within each participant, allowing valid inference without assuming independence across electrodes. Multiple comparisons were corrected using the false discovery rate (FDR) method where applicable (e.g., Figures 3H and 4D) [60]."

C4: *In Figures 2C and D, theta power begins to increase shortly (~50ms) after, and continues to increase up to 200ms after, saccade onset. I appreciate that "saccade durations varied across events" (with a median value of ~90ms), and that later time points are more likely to overlap with fixations, but it would still be interesting to know how long this theta power increase persisted for (particularly for the memory-cued condition).*

Response: We appreciate the reviewer's insightful question regarding how long the theta power increase persists following saccade onset, particularly for the memory-cued condition. To clarify, in Figures 2C and 2D (and the corresponding panels in Figures 3B–C and 3E–F), the data to the right of t = 0 reflect only saccade epochs, whereas the data to the left reflect only fixation epochs. Because saccade durations vary (median = 64 ms), later time points after saccade onset reflect only the subset of longer saccades. Thus, the apparent rise extending to ~200 ms does not indicate a sustained increase into fixation but rather reflects the proportion of saccades that remain ongoing at those time points. To directly evaluate how long theta activity persists relative to saccade offset, we additionally examined data aligned to fixation onset in memory-cued navigation (Figure R2 below). As shown in this figure, theta power increases during the saccade and remains significantly elevated briefly after fixation onset before returning to baseline. This pattern indicates that the post-onset elevation reflects the tail end of saccade-related theta, not a fixation-specific increase. Together, these

analyses demonstrate that the theta enhancement is restricted to the saccade and its immediate aftermath, with no evidence for a prolonged increase during the fixation period itself.

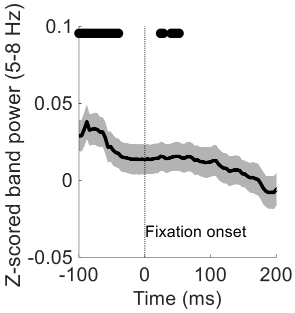

Figure R2. Theta power aligned to fixation onset in memory-cued navigation. Normalized (z-scored) theta band power (5–8 Hz) is plotted around fixation onset (t = 0). The period before fixation onset corresponds to saccades, whereas the period after t = 0 corresponds to fixation epochs. Theta power increases during the saccade and remains significantly elevated for the first few hundred milliseconds after fixation onset (Black horizontal bars = $p < 0.05$) before returning to baseline. This pattern indicates that the post-fixation elevation reflects the decay of saccade-related theta activity rather than a fixation-specific increase. Shaded regions denote ± SEM across channels ($N_{channels}$ = 16).

C5: *At the top of page 8, the authors ask whether saccade related increases in theta power can be explained by other behavioural variables - such as distance from boundaries, body movement etc. In each case, they compare p-values for tests of theta power in each condition. Instead (or in addition to this), they should directly compare saccade related theta power in each condition using a paired t-test (or equivalent) and report those results. In addition, it is not clear to me why separate linear models were fit for each condition - why not have a single mixed linear model and look for any significant interaction?*

Response: We appreciate the reviewer's suggestion to directly compare saccade-related theta power between conditions. As requested earlier (see C2 above), we reanalyzed the data using the PALM framework and performed paired MC–VC contrasts for each behavioral context (boundary, inner region, movement, and stationary), now shown in S5 Fig. Boundary proximity showed a robust MC–VC difference (p = 0.008). For the inner region, movement, and stationary periods, effects did not reach significance, although movement and stationary exhibited modest trends toward stronger saccade-related theta modulation in the memory-cued condition (movement: p = 0.051; stationary: p = 0.058). These results indicate that differences in locomotion or movement state do not account for the theta increases observed in the MC condition. To address the reviewer's suggestion to use a single mixed linear model, we fit a unified linear mixed-effects model that included Condition (MC vs. VC), Epoch (saccade vs. fixation), and the behavioral variables (boundary vs. inner position, locomotion speed, and stationary state), along with their interactions, with random effects for subjects and channels. In this comprehensive model, none of the interactions involving Condition or Epoch, and none of the behavioral interactions reached significance, and the main effects of Condition and Epoch were also non-significant. We believe this reflects reduced sensitivity when aggregating across all behavioral contexts, channels, and participants within a single model, which can obscure within-subject effects that are more sensitively detected using PALM. Because PALM constrains permutations within subjects and preserves the structure of the saccade-aligned data, we view it as the more appropriate approach for evaluating condition-specific modulation, as reflected in S5 Fig. Together, these analyses clarify that locomotion speed does not influence saccade-related theta modulation and that boundary proximity remains the strongest behavioral factor modulating saccade-locked theta during memory-guided navigation. We have revised the Results section accordingly, adding the following text:

Results (pg. 8, lines 190-202):

"We next examined whether saccade-related theta increases were modulated by other behavioral contexts. We quantified z-scored theta power during saccades across four contexts: near boundaries, in inner regions, at higher locomotion speeds (movement), and while stationary (S5A–D Fig). Saccade-related theta increases were significant only near boundaries during memory-cued navigation ($N_{channels}$ = 16, MC: t = 2.62, p = 0.006; VC: t = –0.02, p = 0.506; MC vs. VC: t = 2.51, p = 0.008). In contrast, no significant effects were observed in inner regions for either condition ($N_{channels}$ = 16, MC: t = 1.00, p = 0.163; VC: t = 0.66, p = 0.163; MC vs. VC: t = 0.45, p = 0.324). During movement and stationary periods, effects trended towards significance only during memory-cued navigation (movement: MC: t = 1.60, p = 0.060; VC: t = 0.10, p = 0.462; MC vs. VC: t = 1.70, p = 0.051; stationary: MC: t = 1.35, p = 0.095; VC: t = 0.14, p = 0.440; MC vs. VC: t = 1.62, p = 0.058). Taken together, these results show that the observed increases are not explained by locomotion or general movement state and instead appear most robust near boundaries, where spatial information is especially salient for memory-guided navigation."

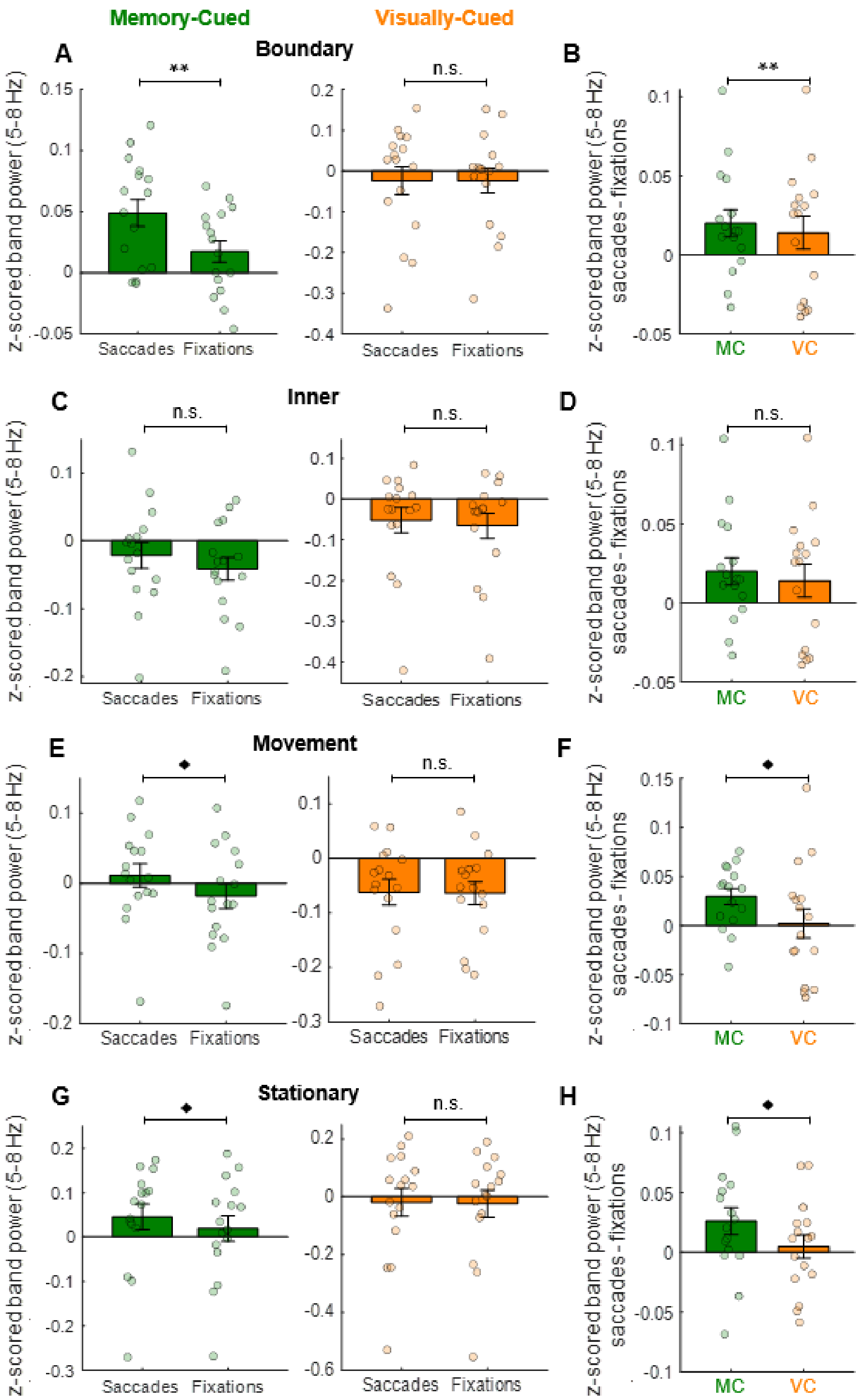

S5 Fig. Control Analyses for Saccade-Related Modulation of Theta Band Power. Normalized (z-scored) MTL theta power during saccades is shown separately for memory-cued and visually-cued conditions under four behavioral contexts: **(A)** near boundaries (< 1.2 m from the wall), **(B)** inner region (> 1.2 m from the wall), **(C)** during movement, and **(D)** while stationary. Saccade-related theta increases were observed primarily during memory-cued navigation, with a significant effect near boundaries and trends in the other contexts. Each point reflects a single MTL channel (N = 16); bars represent mean ± SEM. * = $p < 0.05$, ◆ = $p < 0.01$, n.s. = not significant.

C6: *Finally, I wonder if the authors wish to speculate on the implication of these results for previous reports of movement related MTL theta power. Is it possible that participants make more saccades during movement (when the visual scene changes) than during stationary periods (perhaps the authors could test that in their data?), and that previous reports of more prevalent theta oscillations during movement could therefore be explained by increased saccade frequency, rather than translational movement per se?*

Response: We thank the reviewer for raising this important point. To test whether locomotion might induce more frequent saccades—and thereby account for prior reports of movement-related MTL theta—we compared saccade frequency during movement and stationary periods in our dataset. Saccade rates were statistically indistinguishable across movement and stationary epochs in both conditions (MC: 2.38 Hz vs. 2.36 Hz, $p = 0.392$; VC: 2.12 Hz vs. 2.32 Hz, $p = 0.904$, Figure R3 below). Thus, participants did not make more saccades during locomotion than while stationary. These results suggest that movement-related theta reported in earlier studies is unlikely to be explained by an increase in saccade frequency alone. Instead, our findings point to a different interpretation: when memory is engaged, saccades become more strongly coupled to hippocampal–MTL theta, regardless of whether the participant is moving or stationary. In the visually-cued condition, where memory demands are minimal, this saccade–theta coupling is absent, despite comparable saccade rates. Together, these observations indicate that the critical factor is not the number of saccades occurring during movement, but rather the memory-dependent modulation of theta during saccades.

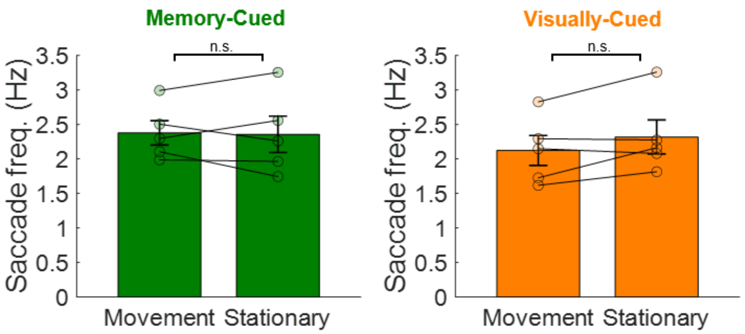

Figure R3. Saccade frequency during movement and stationary periods. Mean saccade rates (Hz) are shown for movement and stationary epochs in the memory-cued and visually-cued navigation conditions. Saccade frequency did not differ between movement and stationary periods in either condition (memory-cued: 2.38 vs. 2.36 Hz, $p = 0.392$; visually-cued: 2.12 vs. 2.32 Hz, $p = 0.904$). Each point represents a participant; bars show mean ± SEM. These results indicate that locomotion does not increase saccade frequency and therefore cannot account for the memory-related enhancement of saccade-locked MTL theta.

Minor Comments

C7: *Introduction: "This contrasts with the approach of directly recording brain activity during freely-moving ambulatory behavior in rodents" - this seems like a repeat of the information in the previous sentence, the authors might consider cutting?*

Response: We thank the reviewer for this suggestion. We agree that this sentence was redundant and have removed it from the revised manuscript.

C8: *Results: "We analyzed mobile iEEG recordings in five individuals who were surgically implanted with the RNS System … In addition, we examined eye and body movements captured while participants were engaged in a spatial navigation task within a rectangular room" - this makes it sounds as if these were two separate cohorts or analyses - perhaps it would be better to say "Alongside the EEG signals, we examined eye and body…" ?*

Response: We thank the reviewer for catching this ambiguity. We have revised the sentence (pg. 4, line 93) to read:

"Alongside the iEEG signals, we examined eye and body movements captured while participants were engaged in a spatial navigation task within a rectangular room (Figure 1C, S1A Fig)." This clarification makes clear that all measures were obtained simultaneously from the same participants.

C9: *Methods: "via computerized to remain still" - possible typo?*

Response: We thank the reviewer for noting this typo. The sentence (pg. 21, line 532) has been corrected and now reads:

"Participants were instructed via computerized audio prompts to remain still."

C10: *Methods: The iEEG data analysis section is confusing, because it briefly describes the BOSC toolbox and method, but as far as I can tell, all results presented in the manuscript use oscillatory power as estimated by a wavelet transform, rather than the prevalence of theta bouts as estimated by BOSC. Can the authors please clarify?*

Response: We thank the reviewer for this helpful clarification request. The reviewer is correct that the majority of analyses use a continuous Morlet wavelet transform to compute oscillatory power, which is implemented within the BOSC toolbox framework. However, we also used the BOSC method specifically to estimate the prevalence of theta bouts, which is shown in S1C Fig for both memory-cued and visually-cued conditions. We

have clarified this distinction in the revised *Methods* section to indicate that wavelet-based power analyses were used for the main results, whereas the BOSC approach was applied for quantifying theta bout prevalence in S1C Fig.

Methods (iEEG Data Analysis section; pg. 22, lines 558-566):

"Time–frequency analyses of iEEG data were conducted using the BOSC toolbox [26,59], which includes both continuous wavelet-based power estimation and detection of oscillatory bouts based on duration and power criteria. For all main analyses, oscillatory power was computed using a continuous Morlet wavelet transform (implemented within the BOSC framework), which estimates time-resolved power at each frequency. Morlet wavelets of order six were applied across frequencies from 2 to 30 Hz in 0.25 Hz steps, and 31 to 80 Hz in 1 Hz steps. To quantify the prevalence of theta bouts, we additionally used the BOSC detection algorithm, which identifies time points where power exceeds the 95th percentile of a fitted background (1/f) distribution for at least two cycles; these results are reported in S1C Fig."

C11: *Figure 4: Do these figures all have y-axis units of Z - i.e. report average Z-scored data? If so, please add the units accordingly (this is more specific than 'normalised' power, which is fairly ambiguous)*

Response: Yes, the y-axes in Figure 4 (and related figures) represent z-scored power values, computed within each channel and frequency band before averaging across electrodes and/or participants. We have updated the figure y-axis labels and captions to specify "z-scored band power (5–8 Hz)" instead of the more general term "normalized power."

C12: *Methods: It would be useful if the authors could report how many trial numbers in each condition were completed by each participant, how many fixation periods were included, and show the distribution of fixation durations (i.e. to match the data for saccades presented throughout the manuscript).*

Response: We thank the reviewer for this helpful suggestion. In the revised manuscript, we now include a supplementary table reporting the number of completed trials as well as total fixation and saccade counts for each participant in each condition (S1 Table).

Participant	Trials Completed (per condition)	Saccades/Fixations (MC)	Saccades/Fixations (VC)
P1	36	3,160	579
P2	125	3,175	1,847
P3	156	4,829	2,170
P4	147	5,426	3,234
P5	151	3,874	1,718

S1 Table. Number of completed trials and saccade and fixation counts per participant and condition. Values indicate the total number of trials, saccades, and fixation periods included in the memory-cued (MC) and visually-cued (VC) conditions for each participant.

In addition, we have added a supplementary figure panel illustrating the distribution of fixation durations (S1B Fig), directly complementing the saccade data presented in the same figure. These additions provide a clearer characterization of the behavioral data underlying our analyses.

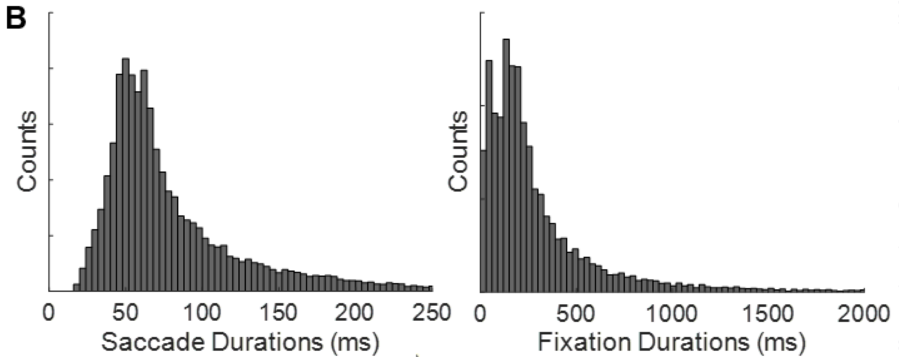

S1 Fig. (B) Distribution of saccade durations (ms) and fixation durations (ms) across all participants ($n_{participants} = 5$).

Reviewer #3: Zubair et al. report the result of the analysis of simultaneously recorded hippocampal LFP and eye movements in freely-moving human participants, during the performance on memory-guided and visually-guided navigation. They report higher theta power (5-8 Hz) during saccadic eye movements, relative to fixation periods, selectively during the trials with memory demands. In addition, hippocampal theta power was associated with spatial memory performance and higher amplitude saccades. The results are interesting and contribute to understanding the primate-specific relation between the visual sampling, memory and navigation. However, some of the aspects of analysis and interpretation deserve scrutiny and thorough clarification, before the paper could be deemed acceptable for publication.

Response: We thank Reviewer #3 for the thoughtful and constructive evaluation of our manuscript and for recognizing the significance of our findings linking visual sampling, memory, and navigation in freely moving humans. We appreciate the reviewer's insightful comments regarding aspects of the analysis and interpretation. In the revised manuscript, we have addressed each point in detail below, adding new analyses and clarifications that strengthen the robustness and interpretability of our results.

Major points:

C1: *The fixation baseline used for contrasting the saccadic vs. fixation theta is -100 - 0 ms, relative to saccadic onset. However, as evident from Fig. 2B, theta power increases over the baseline around 50ms post-saccadic onset and keeps ramping up until 250ms. The crucial aspect missing from this analysis is the time course of theta power after the saccade offset? Since the fixations are typically quite longer than the last 100ms used for this comparison, the baseline used in the analysis might result in generalizing the theta power just prior to saccade onset as characteristic for the entire fixation period. If the theta power remains elevated during the early fixation period, it's hard to argue that the theta is increased during saccades, relative to fixation periods. This question should be clarified by showing the analysis relative to saccade offset, as well. The statement of higher theta during saccade, relative to fixation, should be re-examined in the context of this analysis.*

Response: We thank the reviewer for this important clarification. First, we note that Fig. 2B displays broadband low-frequency power (3–15 Hz), not theta specifically. This panel was intended to illustrate overall low-frequency dynamics around saccade onset, but we agree that the reviewer's question pertains specifically to theta-band activity. Regarding baseline selection, theta power for saccade–fixation comparisons was not normalized using only the −100 to 0 ms pre-saccadic window. Instead, all theta values were z-scored using the mean power across all saccade and fixation periods across both conditions. Thus, the −100 to 0 ms segment in Fig. 2B was shown only for visualization and was not used as the normalization baseline. To directly address the reviewer's question about theta dynamics following saccade offset, we now include a new analysis aligned to fixation onset during memory-cued navigation (**Figure R2**). This analysis shows that theta power peaks during the saccade and remains elevated for roughly the first 50 ms after fixation onset before returning to baseline. As indicated by the statistical markers (black bars indicate $p < 0.05$), this brief post-fixation elevation we believe reflects the decaying tail of saccade-related theta, rather than a fixation-specific increase. We believe these findings reinforce that theta increases are driven by saccades, not by sustained elevations during fixations.

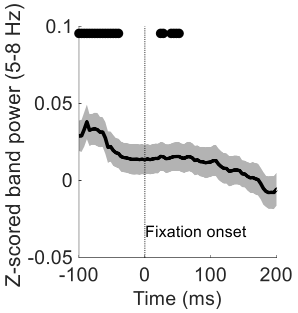

Figure R2. Theta power aligned to fixation onset in memory-cued navigation. Normalized (z-scored) theta band power (5–8 Hz) is plotted around fixation onset (t = 0). The period before fixation onset corresponds to saccades, whereas the period after t = 0 corresponds to fixation epochs. Theta power increases during the saccade and remains significantly elevated for the first few hundred milliseconds after fixation onset (Black horizontal bars = $p < 0.05$) before returning to baseline. This pattern indicates that the post-fixation elevation reflects the decay of saccade-related theta activity rather than a fixation-specific increase. Shaded regions denote ± SEM across channels ($N_{channels} = 16$).

We have clarified our normalization procedure in the revised *Methods* section and updated the Figure 2 caption accordingly:

Figure 2 caption (pg. 6, lines 133-135):

"Normalization (z-scoring) was based on mean theta power across all fixation and saccade periods pooled across conditions, ensuring that contrasts were not biased by short pre-saccadic windows."

Methods (Temporal Analysis section; pg. 25, lines 627-628):

“For normalization, baseline power was defined as the mean theta power across all fixation and saccade periods pooled across both task conditions.”

C2: *As shown in multiple previous publications (e.g. Katz et al., 2022, Fig. 4A), peri-saccadic hippocampal LFP is dominated by the ERP with early peak starting around the same time as the theta increase during saccades in the current study (50 ms post saccadic onset; Fig. 2C in Katz et al., 2020). The authors should acknowledge that the appearance of theta (or broader low-frequency power) in peri-saccadic spectrograms could also originate from this activity.*

Response: We thank the reviewer for this insightful comment and agree that peri-saccadic event-related potentials (ERPs) can contribute to broadband low-frequency power changes in hippocampal LFPs. We have therefore added a statement acknowledging this possibility in the revised *Discussion*. Specifically, we note that the observed peri-saccadic theta increase may partially reflect overlapping ERP components, as reported in previous studies, e.g. (Katz et al., 2020, 2022). However, we also highlight that our analyses focused on **time–frequency power averaged across multiple saccades and fixation periods**, rather than single-trial ERP waveforms, and that similar time-locked increases were observed in the phase-based measure (PPC), supporting the interpretation of rhythmic synchronization rather than purely evoked transients.

Discussion (pg. 15, lines 372-378):

“It is important to note that peri-saccadic low-frequency power increases may partially reflect overlapping event-related potential (ERP) components, as reported in prior work [24,25]. Such evoked responses can produce broadband low-frequency power that temporally coincides with theta activity. Nonetheless, our analyses emphasized time–frequency power averaged across many saccades and fixations, rather than single-trial ERP waveforms, and the presence of clear phase resetting (PPC) effects supports the interpretation that rhythmic synchronization contributes meaningfully to the observed signal.”

C3: *In addition, these previous studies in humans (Katz et al., 2020, 2022) and non-human primates (Hoffman et al., 2013) showed that this peri-saccadic ERP / transient theta is present during visual tasks that do not put demands on memory as in the current analyses. Other work links saccade-locked theta during encoding to subsequent memory (Jutras, Fries & Buffalo, 2013), while the current reported results seem to suggest these signals are present for memory retrieval. Thus, a more in depth treatment of these discrepancies and why the current results deviate from prior literature is warranted. Furthermore, some of these papers emphasized theta phase resetting more than power changes per say. Thus, it is possible that theta phase was reset during non-memory related tasks, even if there was no commensurate change in power. Thus, follow up phase consistency analyses should be performed to rule out this possibility, to better fit with the extant literature. This is especially important considering the theoretical importance of theta phase for memory-perception interactions (Hasselmo, Bodelón & Wyble, 2002; Lisman & Buzsáki, 2008).*

Response: We thank the reviewer for this thoughtful and important comment. We agree that prior work has shown peri-saccadic theta or ERP transients during visually guided tasks without explicit memory demands (e.g., Katz et al., 2020, 2022; Hoffman et al., 2013), and that theta phase resetting plays a critical role in memory–perception interactions (Hasselmo et al., 2002; Lisman & Buzsáki, 2008). To address this point, we performed **phase-consistency analyses** using **pairwise phase consistency (PPC)** to directly test for saccade-locked theta phase alignment in our dataset (Vinck et al., 2010; Hoffman et al., 2013). As described in the revised *Results* (see new S3 Fig), we found **significant theta phase alignment around saccade onset** in both **memory-cued and visually-cued navigation conditions**, indicating that saccade-related phase resetting occurs even in the absence of explicit memory demands. However, the **magnitude and duration of phase alignment were greater in the memory-cued condition**, suggesting that while phase resetting represents a general organizing mechanism during visual sampling, its sustained engagement may depend on mnemonic context. We have expanded the *Discussion* (copied below) to reconcile our findings with prior literature, emphasizing that our data support a continuum in which saccade-related theta phase resetting occurs broadly across tasks, but power modulation and extended phase alignment are most pronounced when retrieval demands are high.

Discussion (pg. 15, lines 365-371):

“We also found evidence of theta phase resetting around saccade onset, consistent with prior work in both humans and non-human primates [2,6,24,25]. Phase resetting was observed in both memory-cued and visually-cued navigation, suggesting that saccade-locked theta serves as a general temporal coordination

mechanism in the MTL. However, the phase resetting persisted longer after saccade onset during memory-cued navigation, potentially reflecting additional mnemonic or planning-related processes. This observation supports models proposing that theta phase alignment provides a temporal framework for organizing perception and memory [20,37]."

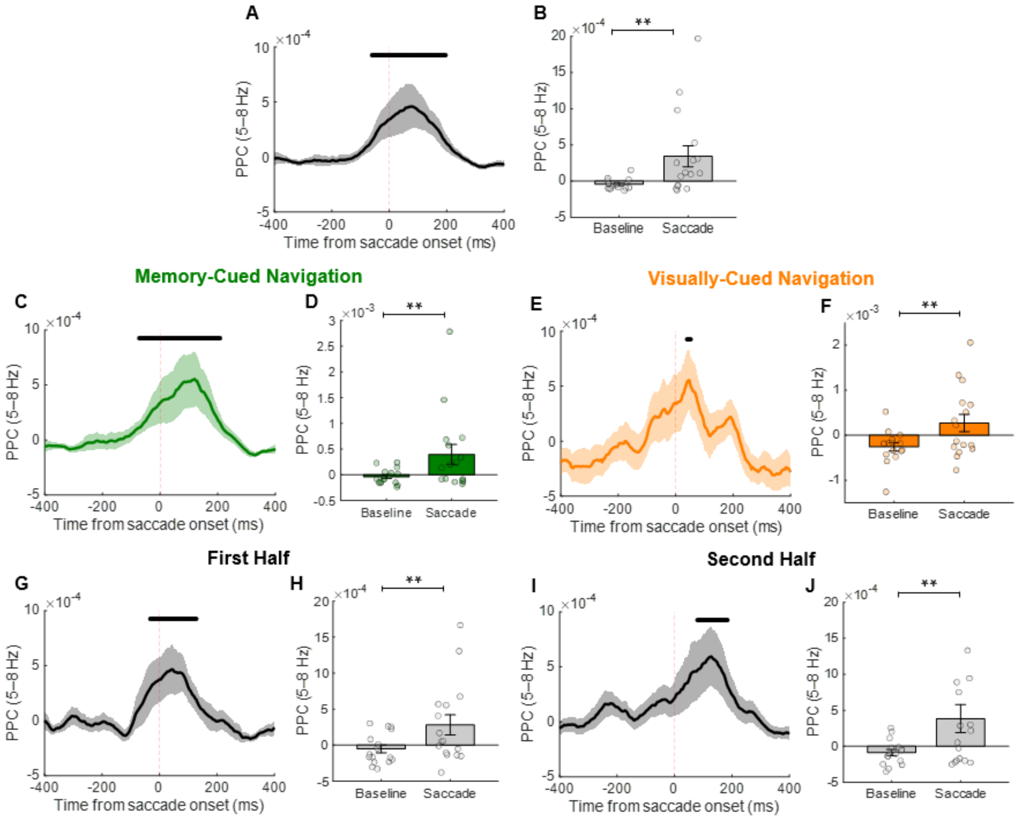

S3 Fig. Theta phase resetting to saccade onset. (A–B) Theta phase resetting relative to saccade onset, quantified using pairwise phase consistency (PPC). PPC was significantly elevated from –60 to +196 ms around saccade onset (black bar = p < 0.05, shaded areas represent SEM across participants; A), and PPC during the saccade-onset window (–50 to +200 ms) was significantly higher than an equivalently sized baseline period (–800 to –550 ms, ** p < 0.01; B). **(C–F)** Time-resolved PPC traces (mean ± SEM across participants, shaded gray) show significant theta phase alignment surrounding saccade onset in both memory-cued (MC) and visually-cued (VC) navigation. **(G–J)** When trials were divided into early and late halves, significant PPC increases were observed around saccade onset in both periods, indicating consistent phase resetting throughout the trial. Bar plots adjacent to each panel display mean PPC during the saccade-onset window (–50 to +200 ms) and a baseline window (–800 to –550 ms). PPC was significantly higher during the saccade period for all comparisons (** p < 0.01). These results demonstrate that theta phase alignment accompanies saccadic events across both task conditions and throughout the course of navigation.

***C4:** Given the median saccade length of 62 ms, the theta doesn't seem to show any increase from baseline for a large fraction of saccades. The results seem to be driven mostly by a long tail of saccades longer than 100 ms. This observation should receive more in depth treatment, when contextualizing the interpretation of the current findings.*

Response: We thank the reviewer for this insightful comment. We agree that saccade duration and magnitude are important factors influencing peri-saccadic neural activity. In line with the reviewer's observation, our analyses revealed that **theta power increases were most prominent during larger-magnitude saccades** (Figure 4), consistent with those lasting longer than approximately 64 ms. This relationship was statistically significant (p = 0.002, indicating that theta modulation scales with saccade amplitude, rather than being uniformly present across all saccades. We have expanded the *Results* and *Discussion* sections to clarify that the observed theta increases primarily reflect **large, exploratory saccades**, which are more likely to engage spatial updating and mnemonic processes. Smaller saccades, by contrast, may contribute less to theta modulation, consistent with their lower behavioral relevance for navigation and memory retrieval.

Discussion (pg. 15, lines 379-388):

"Theta activity was not uniform across the course of a trial. Saccade-related theta power increases were stronger during the early, planning-dominant phase of memory-cued navigation than during later execution.

Moreover, periods of higher scanpath entropy, reflecting more exploratory and dispersed gaze behavior, were associated with greater theta power. The link between exploratory viewing and theta echoes prior work showing that hippocampal theta in both humans and monkeys increases during active visual search [2,38]. This pattern suggests that theta may help organize internal predictions or spatial hypotheses before movement, supporting a transition from memory recall to forward-looking planning. In this view, hippocampal theta could act as a bridge between retrieving past experiences and simulating future trajectories, linking perception, attention, and memory into a cohesive planning process."

C5: *It's difficult to conceptualize that the theta power increases for a relatively small fraction of an oscillatory cycle, which would result either in a highly asymmetric oscillatory waveform, which could again be interpreted as a nested higher frequency oscillations showing the phase-amplitude coupling with underlying theta. The authors do specify that the BOSC toolbox detects oscillations based on duration (greater than or equal to two cycles), and their analyses were also robust even after using wavelets with 3 cycles instead of 6. However, one limitation of wavelet-based signal processing approaches is that, even with a low number of cycles that maximize temporal resolution, they induce temporal smoothing in the signal over time, which can inflate oscillation duration estimates present in the time domain. Thus, it would be informative to see if multicycle rhythms are observed when evaluating rhythms using non-spectral oscillation analysis methods performed in the time-domain, like cycle-by-cycle analysis for example (Cole & Voytek, 2019).*

Response: We appreciate the reviewer's insightful comments regarding oscillatory waveform asymmetry and the potential advantages of time-domain, cycle-by-cycle analyses. Our primary analysis of theta power was conducted using the BOSC algorithm, which estimates narrowband power through Morlet wavelet convolution. While this approach introduces some temporal smoothing, it does not explicitly detect or count oscillatory episodes. Instead, BOSC identifies time points where instantaneous power exceeds a background 1/f threshold, enabling consistent estimation of theta-band activity across the continuous signal. We agree that time-domain methods, such as the cycle-by-cycle (bicycle) approach proposed by Cole and Voytek (2019), provide valuable complementary information by directly quantifying oscillatory amplitude and shape from the broadband waveform without relying on spectral decomposition. To address this suggestion, we performed an exploratory cycle-by-cycle analysis of the same data to examine rhythmic activity in the theta range (Figure R4 below). While this method did not reveal statistically significant differences between saccades and fixations, the direction of effects was consistent with our BOSC- and wavelet-based analyses, showing numerically higher theta amplitude during saccades. These results suggest that although the cycle-by-cycle method may have reduced sensitivity given the short duration and variability of saccades, the overall trend aligns with our frequency-domain findings. We have chosen not to include these exploratory results in the manuscript to maintain focus on the primary spectral analyses, which are more directly suited to capturing transient, narrowband rhythmic changes. Importantly, our conclusions concern differences in theta-band power between cognitive conditions, not the duration or prevalence of multicycle rhythmic bouts. The absence of clear multicycle oscillations in the time domain therefore does not contradict our findings; rather, it underscores that the observed effects are frequency-specific and not driven by broadband or nonspecific amplitude changes. Moreover, the temporal smoothing inherent in BOSC would be expected to dilute rather than exaggerate saccade-related differences, since the brief saccadic events (<100 ms) are averaged with neighboring fixation periods. Thus, the persistence of significant theta increases under these conservative conditions supports the robustness of our findings.

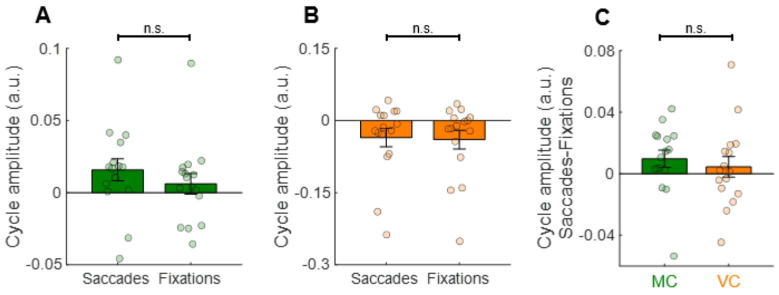

Figure R4. Cycle-by-cycle theta amplitude during saccades and fixations across conditions. Cycle-by-cycle analyses were used to quantify theta amplitude directly from the time-domain signal (Cole & Voytek, 2019). **(A)** In the memory-cued (MC) condition, theta cycle amplitude was numerically higher during saccades than fixations, though not statistically significant. **(B)** In the visually-cued (VC) condition, no difference was observed between saccades and fixations. **(C)** Comparison of the saccade–fixation theta amplitude

difference between conditions showed a numerically greater effect in the MC condition relative to VC, but this difference was not significant. Error bars represent SEM across participants.

C6: Finally, as acknowledged by the authors (Lines 281-286), previously published results from the same group show the correlation between theta power and body movement speed, which is missing in hereby presented results. This should be clarified.

Response: We thank the reviewer for this important point. As noted, our previous studies demonstrated a relationship between theta power and movement speed, but only during non-mnemonic or visually guided navigation tasks. Specifically, Aghajan et al. (2017) showed that theta power scaled with movement speed during a non-mnemonic walking task in which participants simply walked across a room or in circles at different speeds, without memory demands. Likewise, Stangl et al. (2021; Fig. 4E) reported significant modulation of low-frequency power by movement speed during visually guided ("no target search") but not memory-cued ("target search") navigation. The current study analyzes the same dataset as Stangl et al. (2021), focusing here on eye movement–related theta dynamics. Consistent with those prior results, we observed speed did not significantly explain variance in theta power, whereas eye movements did (S4C Fig). This pattern aligns with our more recent memory-guided studies (Maoz et al., 2023; Seeber et al., 2025), which similarly found no significant relationship between theta and speed under mnemonic demands. Together, these findings suggest that theta–speed coupling is a hallmark of non-mnemonic or visually-guided locomotion, whereas theta activity during memory-guided navigation primarily reflects internal cognitive processes such as retrieval and planning, rather than external movement parameters. We have clarified this distinction in the revised *Discussion* section.

Discussion (pg. 15, lines 389-401):

"Previously, we showed that MTL theta is modulated by spatial location, movement speed, and behavioral demands [1,10,16]. In those studies, walking speed was associated with theta increases only when memory was not explicitly engaged. Aghajan et al. [10] reported that theta prevalence increased with movement speed during a simple walking task without memory demands, and Stangl et al. [16] found significant modulation of low-frequency power by speed during visually-guided ("no target search") but not memory-cued ("target search") navigation. Using the same dataset as Stangl et al. [16], the present study focuses on eye movement–related theta dynamics and similarly finds that movement speed did not significantly explain variance in theta power, whereas eye movements did (S4C Fig). This pattern is consistent with our more recent memory-guided studies [1,39], which also found no relationship between theta and speed under mnemonic conditions. Together, these findings indicate that theta–speed coupling is characteristic of non-mnemonic or visually-guided locomotion, whereas during memory-guided navigation, theta dynamics reflect internal cognitive processes such as retrieval and planning rather than external movement parameters."

Minor points:

C7: *It's unclear if the trial type (memory vs. visually-guided) was alternated following each trial or if there were longer blocks of the same trial type.*

Response: We thank the reviewer for this comment and note that the trial structure is described in the *Methods* section. Specifically, the trial type alternated between memory-cued and visually guided navigation, as stated (pg. 19, lines 476-479): "Trials alternated between two types: (1) visually-cued navigation, where participants walked to a visible wall sign (e.g., 'yellow 4'), and (2) memory-cued navigation, where they searched for a previously learned but currently hidden target location (e.g., 'T')."

C8: *Were the subjects given any feedback about how close their final position was to the invisible target?*

Response: Participants received auditory feedback when they entered the 0.7 m diameter invisible target area, indicating successful arrival at the target location. This auditory cue allowed participants to gradually learn and remember the target locations through experience. We have clarified this detail in the revised *Methods* section (pg. 19, lines 479-482):

"Hidden targets were not shown prior to the task. Instead, during initial trials, participants were instructed to explore until they reached the correct location, at which point an audio tone signaled that they were within the target's 0.7 m radius. This auditory feedback was repeated each time the target was reached, strengthening memory across trials."

C9: *Were the different targets randomly selected in consecutive memory trials or there were blocks when the participants searched for the same targets?*

Response: Participants performed the task in blocks in which they searched for the same set of target locations. Within each block, trials alternated between visually guided and memory-cued navigation to these same targets, but the order of target presentation was randomized across blocks. We have clarified this detail in the revised *Methods* section (pg. 19, lines 482-485):

"Each participant completed multiple blocks of alternating trial types (2–4 blocks, ~15 min each). The same hidden targets were repeated within a block (four for Participant 1; three for Participants 2–5), and the order of targets varied across blocks."

C10: *It is unclear how the trials were divided into low-, medium- and high-performance. Was it based on clustering or data points split into equal-sized thirds or some other objective method?*

Response: Trials were divided into low-, medium-, and high-performance trials by splitting the data into equal-sized thirds based on behavioral performance scores. We have clarified this in the revised *Methods* section (pg. 19, lines 488-490):

"Trials were divided into low-, medium-, and high-performance trials by splitting the data into equal-sized thirds based on detour error scores."

C11: *It would be interesting to know if the theta during saccades increased specifically during saccades that landed on the target location.*

Response: We appreciate this thoughtful suggestion. To address it, we classified each saccade according to whether it brought the gaze closer to the upcoming goal location ("goal-convergent") or shifted it farther away ("goal-divergent"). We then compared saccade-related theta modulation across these categories. Although theta increases were numerically larger for goal-convergent saccades, this effect was not statistically significant across channels ($N_{channels} = 16$, $t = 0.586$, $p = 0.273$, Figure R1 below). Although we do not incorporate this analysis into the manuscript, we believe this remains an interesting question that future studies with larger datasets should explore further.

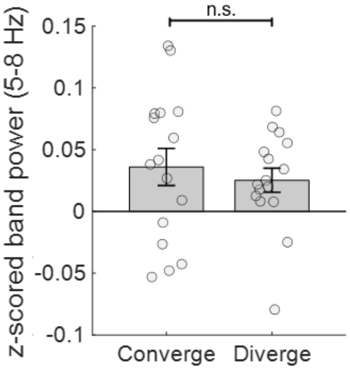

Figure R1. Theta modulation during goal-convergent vs. goal-divergent saccades. Saccades were classified based on whether they moved gaze closer to (convergent) or farther from (divergent) the upcoming goal. Although convergent saccades showed slightly higher mean theta activity, the effect was not significant across channels ($t = 0.586$, $p = 0.273$). n.s. = not significant.

References

M. Aghajan Z, Schuette P, Fields TA, Tran ME, Siddiqui SM, Hasulak NR, et al. Theta Oscillations in the Human Medial Temporal Lobe during Real-World Ambulatory Movement. Current Biology. 2017;27: 3743-3751.e3. doi:10.1016/j.cub.2017.10.062

Cole S, Voytek B. Cycle-by-cycle analysis of neural oscillations. Journal of Neurophysiology. 2019;122: 849–861. doi:10.1152/jn.00273.2019

Harris RL, Glover B, Spady A. Analytical Techniques of Pilot Scanning Behavior and Their Application. NASA Technical Paper. 1986;2525: 1–41.

Hasselmo ME, Bodelón C, Wyble BP. A Proposed Function for Hippocampal Theta Rhythm: Separate Phases of Encoding and Retrieval Enhance Reversal of Prior Learning. Neural Computation. 2002;14: 793–817. doi:10.1162/089976602317318965

Hoffman KL, Dragan MC, Leonard TK, Micheli C, Montefusco-Siegmund R, Valiante TA. Saccades during visual exploration align hippocampal 3–8 Hz rhythms in human and non-human primates. Front Syst Neurosci. 2013;7. doi:10.3389/fnsys.2013.00043

Jutras MJ, Fries P, Buffalo EA. Oscillatory activity in the monkey hippocampus during visual exploration and memory formation. Proc Natl Acad Sci USA. 2013;110: 13144–13149. doi:10.1073/pnas.1302351110

Katz CN, Schjetnan AGP, Patel K, Barkley V, Hoffman KL, Kalia SK, et al. A corollary discharge mediates saccade-related inhibition of single units in mnemonic structures of the human brain. Current Biology. 2022;32: 3082-3094.e4. doi:10.1016/j.cub.2022.06.015

Katz CN, Patel K, Talakoub O, Groppe D, Hoffman K, Valiante TA. Differential Generation of Saccade, Fixation, and Image-Onset Event-Related Potentials in the Human Mesial Temporal Lobe. Cerebral Cortex. 2020;30: 5502–5516. doi:10.1093/cercor/bhaa132

Lisman J, Buzsaki G. A Neural Coding Scheme Formed by the Combined Function of Gamma and Theta Oscillations. Schizophrenia Bulletin. 2008;34: 974–980. doi:10.1093/schbul/sbn060

Maoz SLL, Stangl M, Topalovic U, Batista D, Hiller S, Aghajan ZM, et al. Dynamic neural representations of memory and space during human ambulatory navigation. Nat Commun. 2023;14: 6643. doi:10.1038/s41467-023-42231-4

Melnyk K, Friedman L, Komogortsev OV. What can entropy metrics tell us about the characteristics of ocular fixation trajectories? Wollstadt P, editor. PLoS ONE. 2024;19: e0291823. doi:10.1371/journal.pone.0291823

Seeber M, Stangl M, Vallejo Martelo M, Topalovic U, Hiller S, Halpern CH, et al. Human neural dynamics of real-world and imagined navigation. Nat Hum Behav. 2025;9: 781–793. doi:10.1038/s41562-025-02119-3

Stangl M, Topalovic U, Inman CS, Hiller S, Villaroman D, Aghajan ZM, et al. Boundary-anchored neural mechanisms of location-encoding for self and others. Nature. 2021;589: 420–425. doi:10.1038/s41586-020-03073-y

Vinck M, Van Wingerden M, Womelsdorf T, Fries P, Pennartz CMA. The pairwise phase consistency: A bias-free measure of rhythmic neuronal synchronization. NeuroImage. 2010;51: 112–122. doi:10.1016/j.neuroimage.2010.01.073

Winkler AM, Ridgway GR, Webster MA, Smith SM, Nichols TE. Permutation inference for the general linear model. NeuroImage. 2014;92: 381–397. doi:10.1016/j.neuroimage.2014.01.060
