# Supplementary material for: Eye movements reflect memory-related theta activity in the human brain
Source: PLoS Biol. 2026 Mar 16;24(3):e3003695. doi: 10.1371/journal.pbio.3003695 (PMC13004526; doi:10.1371/journal.pbio.3003695)
Supplement: Attachment — Submitted filename: Response_to_Reviewers_auresp_3.pdf [file pbio.3003695.s013.pdf]

Response to Reviewers

Dear Reviewers,

Response: We thank the reviewers for their careful reading of our manuscript and for the constructive, insightful feedback provided across rounds of review. We have revised the manuscript to add new analyses, clarify methodological details, and refine the interpretation of the findings based on this feedback. These changes strengthen the rigor of the work and sharpen its conceptual contributions regarding the relationship between eye movements, theta dynamics, and internally driven cognition during naturalistic navigation. Below, we provide a point-by-point response to each comment, with reviewer comments reproduced in italics followed by our responses and corresponding changes to the manuscript.

Reviewer #1:

I appreciate the authors' thoughtful, and technically rigorous responses to my comments. They have comprehensively addressed my concerns with substantial new analyses that significantly strengthen the manuscript and sharpen its conceptual contributions. In particular, the new analyses dissociating planning and execution phases provide important clarification. Demonstrating that saccade-related theta modulation is strongest during the early, planning-dominant phase of memory-guided navigation extends the interpretation beyond memory retrieval alone. The addition of scan path entropy analyses further reinforces this conclusion by linking increased theta power to more exploratory and dispersed gaze patterns specifically during memory-guided navigation. Together, these findings position theta as a neural signature of internally driven planning during active sensing, broadening its functional role beyond memory per se. Equally important, the demonstration of robust theta phase resetting aligned to saccade onset, present across both memory-guided and visually guided conditions and throughout trial epochs, supports the idea of saccades acting as temporal anchors for MTL network coordination. The fact that phase resetting occurs in both paradigms argues for a general organizing mechanism coupling visual sampling with hippocampal timing.
Overall, the authors have responded constructively and comprehensively, adding multiple converging analyses that elevate both the rigor and the interpretive clarity of the work. The revised manuscript represents an important advancement in our understanding of how eye movements, theta dynamics, and internal cognitive processes such as planning and memory interact during naturalistic human behavior.

Response: We thank the reviewer for their generous and thoughtful comments. We appreciate their recognition that the new analyses strengthen the rigor and sharpen the conceptual contributions of the manuscript, particularly in clarifying the role of theta in planning and in highlighting saccades as a general temporal organizing mechanism for MTL dynamics.

Reviewer #2*:*

Comment 1 [C1]: *Results (relating to my previous comment C3): It might be clearer if the description of the PALM framework is moved one sentence earlier in the Results, so that it appears just before the first statistical result that is generated using that framework, eg: "Channel-level statistics across the five participants were computed using the Permutation Analysis of Linear Models (PALM) framework [17], which constrains permutations within participants. This approach maintains appropriate statistical independence while preventing effects from being driven by any single individual. Because PALM operates at the level of individual channels while preserving participant structure, we report channel-level analyses throughout the manuscript. Using this approach, we observed a significant increase in MTL power within the low-frequency range (~3-15Hz) during saccades compared to fixation periods when aggregating across channels (Nchannels = 16; t = 3.71, p < 0.001; Figure 2A-B). Additionally, we found that low-frequency power rose gradually following saccade onset..."*

Response: We thank the reviewer for this helpful suggestion. We agree that introducing the PALM framework earlier improves clarity, and we have revised the **Results** section to introduce the PALM analysis immediately before the first statistical results derived from this approach, making the statistical framework explicit prior to the associated findings.

Results (pg. 5, lines 114-121):

“Channel-level statistics across the five participants were computed using the Permutation Analysis of Linear Models (PALM) framework [17], which constrains permutations within participants. This approach maintains appropriate statistical independence while preventing effects from being driven by any single individual.

Because PALM operates at the level of individual channels while preserving participant structure, we report channel-level analyses throughout the manuscript. Using this approach, we observed a significant increase in MTL power within the low-frequency range (~3-15 Hz) during saccades compared to fixation periods when aggregating across channels ($N_{channels}$ = 16; t = 3.71, p < 0.001; Figure 2A-B)."

Comment 2 [C2]: *Results (relating to my previous comment C5): The various findings relating saccades to theta power remain difficult to interpret - specifically, saccade related theta power during memory guided navigation trials is higher: (i) when participants are close to the boundaries; (ii) during high or medium performance trials; (iii) during the first half of each trial ('planning' period); and (iv) when saccades are faster, longer or more complex. As I described previously, the problem is that each of these contrasts have been performed separately, rather than within a single statistical framework, so it is not clear whether these predictors are correlated or confounded. For example, do memory guided trials tend to start near the boundaries (following navigation to a visual landmark on the wall), meaning that the early phases of navigation tend to take place near the boundaries, explaining (i) and (iii)? Do high and medium performance trials tend to involve faster or more complex saccade patterns, explaining (ii) and (iv)? I am aware that the authors compared saccade duration, frequency, and displacement between trials with different levels of performance - but not speed or complexity. In any case, I appreciate that a linear mixed-effects model may not be suitable in this case, due to the limited sample size, but perhaps the authors could directly examine the relationship between the predictors themselves instead? i.e. can they check whether more time is spent close to the boundaries, or whether saccades are faster, longer, or more complex during the first vs second half of trials, and so on? This might help to clarify the key behavioural parameters that dictate saccade related theta power increases*

Response: We thank the reviewer for raising this important interpretive concern and for the constructive suggestion to directly examine potential relationships among the behavioral predictors. In response, we performed targeted control analyses to assess whether the factors associated with increased saccade-related theta power were correlated or systematically confounded. As now described in the **Results** (pg. 10) and shown below in **Figure R1**, memory-cued trials typically began near the boundary, consistent with the task structure following visually guided navigation. We thus observed a significant increase in the proportion of time spent near the boundary during the first compared to the second half of memory-cued navigation, as well as slightly longer saccade durations in the first versus second half of trials. Importantly, no other behavioral measures differed across these contrasts. Eye speed, scanpath entropy, and boundary occupancy did not differ across performance levels, and eye speed and entropy did not differ between early and late trial phases. Likewise, saccade frequency, saccade displacement, and fixation duration were comparable across trial halves. Eye speed and scanpath entropy also did not differ between boundary and inner regions of the room. Taken together, these analyses indicate that increased boundary proximity and early trial phase are partially linked by task structure, such that early memory-guided navigation typically occurs near environmental boundaries. We therefore interpret the boundary-related and early planning-related theta effects as reflecting overlapping behavioral contexts rather than independent or competing mechanisms. Importantly, the presence of enhanced saccade-related theta during the first half of trials cannot be fully explained by boundary proximity alone, as other eye movement features associated with planning, such as scanpath entropy and eye speed, did not differ between early and late trial phases, nor across boundary conditions. These findings argue against a single behavioral confound driving the observed effects and instead support the interpretation that saccade-related theta modulation reflects internally driven planning processes that are most prominent early in memory-guided navigation and often occur near salient environmental boundaries.

Results (pg. 10, lines 237-255):

"To assess whether observed saccade-related theta effects could be explained by systematic covariation among behavioral predictors, we directly examined relationships between trial phase, spatial context, performance, and eye movement metrics during memory-cued navigation. As expected from task structure, memory-cued trials typically began near the boundary, immediately following visually-cued navigation. Accordingly, participants spent a greater proportion of time near the boundary during the first compared to the second half of memory-cued trials ($N_{channels}$ = 16; t = 3.91, p = 0.032). Saccade duration was also modestly longer during the first versus second half ($N_{channels}$ = 16; t = 2.92, p = 0.032). These results indicate that early trial phase and boundary proximity are partially coupled in this task. Importantly, no other behavioral measures differed across these contrasts. Eye speed, scanpath entropy, and boundary occupancy did not differ across performance levels (all p > 0.19). Eye speed and scanpath entropy also did not differ between early and late

trial phases (all $p > 0.25$), and saccade frequency, saccade displacement, and fixation duration were comparable between the first and second halves of memory-cued navigation (all $p > 0.15$). When comparing boundary and inner regions of the room, eye speed and scanpath entropy did not differ (all $p > 0.09$). Together, these analyses indicate that while early trial phase and boundary proximity co-occur due to task structure, the broader pattern of saccade-related theta modulation cannot be explained by a single behavioral confound or shared covariance among eye movement parameters, consistent with a role for internally driven planning processes that are most prominent early in memory-guided navigation."

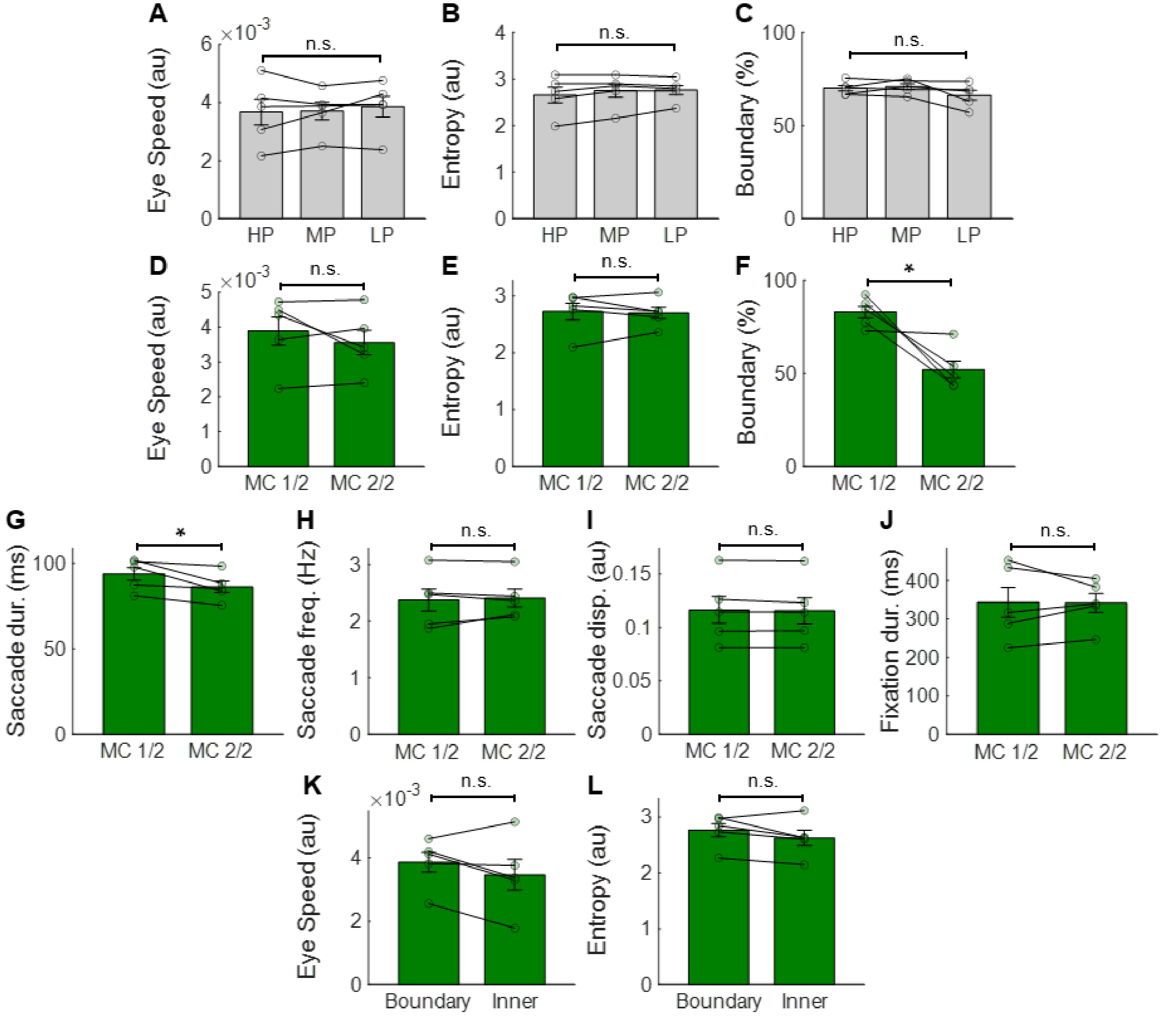

Figure R1. Behavioral control analyses relating performance, trial phase, spatial position, and eye movement metrics during memory-cued navigation. (A–C) Eye speed **(A)**, scanpath entropy **(B)**, and proportion of time spent near the boundary **(C)** shown for low-, medium-, and high-performance memory-cued trials. None of these measures differed significantly across performance levels. **(D–F)** Eye speed **(D)**, scanpath entropy **(E),** and boundary proximity **(F)** for the first versus second half of memory-cued trials. Boundary proximity was significantly greater during the first half of trials, consistent with task structure, whereas eye speed and entropy did not differ across trial halves. **(G–J)** Saccade duration **(G)**, saccade frequency **(H)**, saccade displacement **(I)**, and fixation duration **(J)** for the first versus second half of memory-cued trials. Saccade duration was modestly longer during the first half, while all other eye movement measures were comparable across trial halves. **(K-L)** Eye speed **(K)** and scanpath entropy **(L)** did not differ between boundary and inner regions of the room. Error bars indicate ± SEM. * $p < 0.05$; n.s. = not significant.

Comment 3 [C3]: *Also I believe the key for the significance values in the caption of Figure S5 is wrong: ** is not defined, and the diamond should be p<0.1 rather than p<0.01?*

Response: We thank the reviewer for flagging this. After re-checking the figure captions, we confirmed that the significance key in **S5 Fig.** is correct as currently written. However, we did identify an omission in the caption for **S4 Fig.**: the diamond symbol was not defined. We have now updated the **S4 Fig.** caption to include the diamond as indicating trend-level significance ($p < 0.1$). We also reviewed the significance keys across the remaining supplementary figure captions to ensure consistency and corrected any minor formatting issues where needed.

Comment 4 [C4]: *Results: "standard wavelet approach with a wavenumber of 6" - it might be clearer for a general audience if the authors refer to a "six cycle wavelet approach", as 'wavenumber' is simply a variable name in a specific implementation of the wavelet transform (ie the BOSC toolbox), and I'm not sure there's anything particular standard about choosing a six cycle wavelet, it just happens to be the default parameter in that toolbox. In addition, it would be better to replace 'wavenumber' with 'number of cycles' throughout the remainder of this section. Finally, for the sake of completeness, the authors should also report the results of the other contrasts listed above (i, iii, iv) when a shorter wavelet is used, whether or not they are significant*

Response: We thank the reviewer for this helpful clarification. We agree that referring to a "six-cycle wavelet approach" is clearer and more accessible than using the implementation-specific term "wavenumber." Accordingly, we have replaced "wavenumber" with "number of cycles" throughout the relevant sections of the manuscript and revised the text to describe the analysis as a six-cycle wavelet approach (Results, pg. 14, lines 339-342**)**. In addition, to address the reviewer's request for completeness, we confirmed that the key contrasts reported in the main text remain qualitatively unchanged when using a shorter (three-cycle) wavelet, and these results are now explicitly reported in **S6 Fig.** and in the Results section. Together, these revisions improve clarity for a general audience while demonstrating that the main findings do not depend on the specific wavelet parameter choice.

Results (pg. 14, lines 351-367):

"When comparing high- versus low-entropy periods in MC navigation, theta power was elevated during memory-cued navigation ($N_{channels} = 16$, $t = 2.06$, $p = 0.023$) but not visually-cued navigation ($N_{channels} = 16$, $t = 1.03$, $p = 0.158$; S6D Fig). Similarly, theta power was elevated during long versus short saccades exclusively in memory-cued navigation ($N_{channels} = 16$, $t = 2.71$, $p = 0.004$) but not visually-cued navigation ($N_{channels} = 16$, $t = 0.84$, $p = 0.205$; S6E Fig). Comparing high- with low-frequency saccade periods revealed a trending increase in memory-cued navigation ($N_{channels} = 16$, $t = 1.33$, $p = 0.096$) and no increase during visually-cued navigation ($N_{channels} = 16$, $t = -0.34$, $p = 0.630$; S6F Fig).

Moreover, we confirmed that in boundary regions, MTL theta power was elevated during saccades compared to fixations during memory-cued navigation ($N_{channels} = 16$, $t = 2.60$, $p = 0.007$) but not during VC navigation ($N_{channels} = 16$, $t = -0.02$, $p = 0.506$; S6G Fig). For inner regions of the room, theta power did not differ between saccades and fixations for either memory-cued ($N_{channels} = 16$, $t = 1.16$, $p = 0.130$) or visually-cued navigation ($N_{channels} = 16$, $t = 1.01$, $p = 0.164$; S6H Fig). Finally, comparing theta power between saccades and fixations revealed elevations during the first half of MC trials ($N_{channels} = 16$, $t = 2.29$, $p = 0.013$) but not during the second half ($N_{channels} = 16$, $t = 0.69$, $p = 0.251$; S6I Fig)."

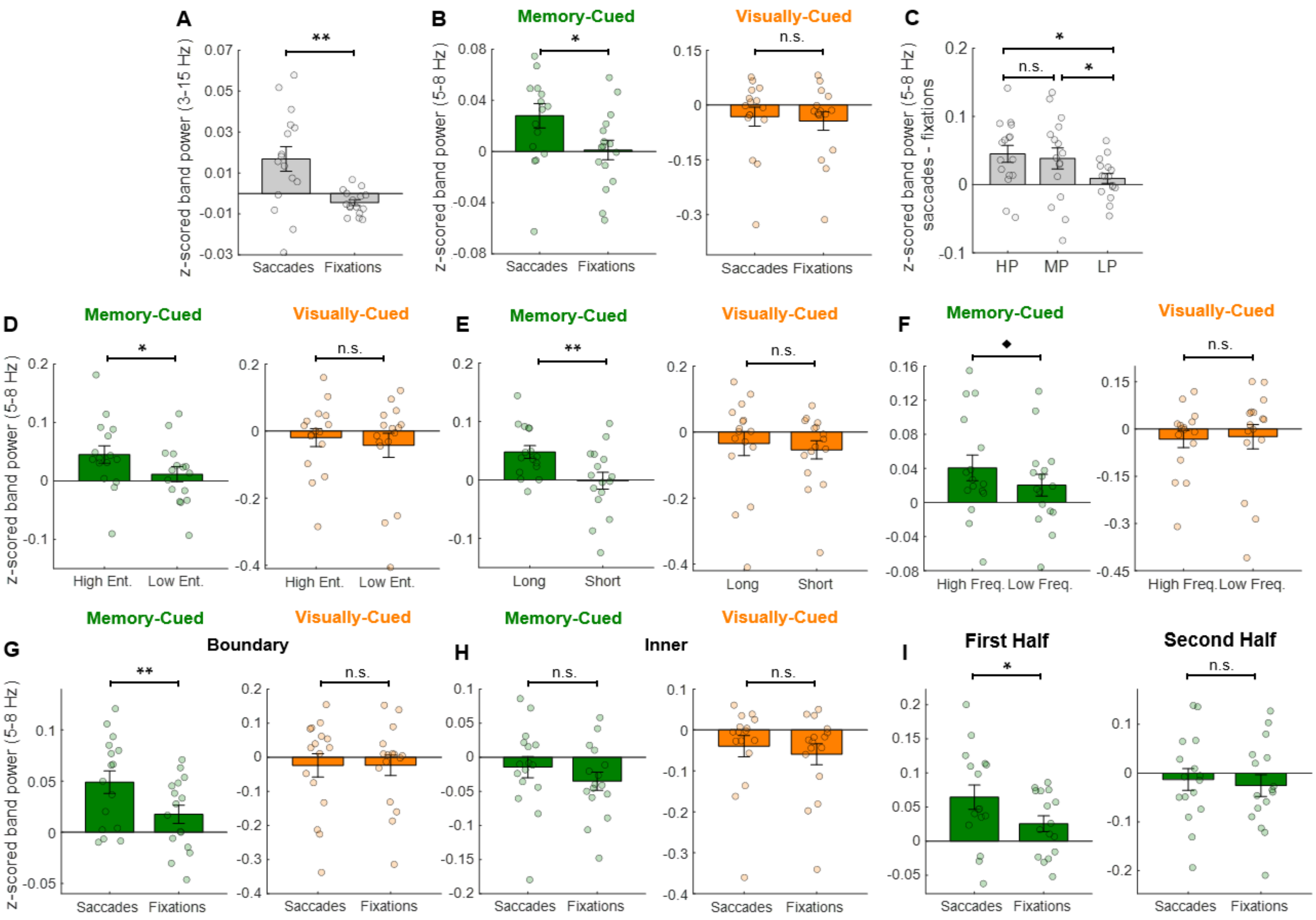

S6 Fig. Analyses with Increased Temporal Resolution. All primary analyses were repeated using BOSC parameters optimized for higher temporal resolution at the expense of frequency resolution (see Materials and Methods). The results remained consistent with the main findings. **(A)** MTL low-frequency (3–15 Hz) power was significantly higher during saccades than fixations (p = 0.001), replicating Figure 2B. **(B)** During memory-cued (MC) navigation, MTL theta (5–8 Hz) power increased during saccades relative to fixations (p = 0.013), replicating Figure 3A; no significant theta increase was observed during visually-cued (VC) navigation (p = 0.258), consistent with Figure 3D. **(C)** Within MC trials, theta power increases were stronger during high-performance (HP; p = 0.012) and medium-performance (MP; p = 0.035) trials compared to low-performance (LP) trials, consistent with Figure 3H. **(D)** Theta power was higher during high- versus low-entropy periods in MC navigation (p = 0.023) but not during VC navigation (p = 0.158) , replicating Figure 4E-F. **(E)** Theta power was higher during long versus short saccades in MC navigation (p = 0.004) but not during VC navigation (p = 0.205), replicating Figure 4B. **(F)** Theta power showed a trend toward being higher during high- versus low-frequency saccades in MC navigation (p = 0.096) but not during VC navigation (p = 0.630), replicating Figure S4D. **(G)** Theta power was higher during saccades compared to fixations in boundary regions during MC navigation (p = 0.007) but not during VC navigation (p = 0.506), replicating Figure S5A. **(H)** Theta power did not differ between saccades and fixations in inner-room regions for either MC or VC navigation (MC, p = 0.130; VC, p = 0.164), replicating Figure S5C. **(I)** Theta power was higher during saccades compared to fixations during the first half of MC trials (p = 0.013) but not during the second half (p = 0.251), replicating Figure S2A-B. All panels show mean ± SEM across 16 MTL channels (circles). * $p < 0.05$, ** $p < 0.01$, ◆ $p < 0.1$; n.s. = not significant.

Comment 5 [C5]: *Discussion: "Importantly, this saccade-theta relationship persisted regardless of the participant's position..." - is this statement true? In the results, the authors show that the saccade-theta relationship is not present in the inner regions of the environment. Please clarify*

Response: We thank the reviewer for catching this inconsistency. This statement was a carryover from an earlier version of the manuscript and is no longer accurate given the revised results demonstrating boundary- and planning-related effects. We have therefore removed this portion of the sentence. The revised text now reads (Results, pg. 15, line 374-376):

“Importantly, this saccade–theta relationship persisted regardless of the participant’s locomotion state, suggesting that eye movements, rather than physical movement, were the primary behavioral correlate of memory-related theta in this context.”

This revision aligns the Discussion with the updated Results.
